# Azobenzene-based inhibitors of human carbonic anhydrase II

**DOI:** 10.3762/bjoc.11.127

**Published:** 2015-07-07

**Authors:** Leander Simon Runtsch, David Michael Barber, Peter Mayer, Michael Groll, Dirk Trauner, Johannes Broichhagen

**Affiliations:** 1 Department of Chemistry, Ludwig-Maximilians-University Munich and Munich Center for Integrated Protein Science, Butenandtstrasse 5–13, 81377 Munich, Germany; 2 Department of Biochemistry, Technical University Munich and Munich Center for Integrated Protein Science, Lichtenbergstr. 4, 85748 Garching, Germany

**Keywords:** azobenzene chemistry, enzyme inhibitors, human carbonic anhydrase II, sulfonamide, X-ray crystallography

## Abstract

Aryl sulfonamides are a widely used drug class for the inhibition of carbonic anhydrases. In the context of our program of photochromic pharmacophores we were interested in the exploration of azobenzene-containing sulfonamides to block the catalytic activity of human carbonic anhydrase II (hCAII). Herein, we report the synthesis and in vitro evaluation of a small library of nine photochromic sulfonamides towards hCAII. All molecules are azobenzene-4-sulfonamides, which are substituted by different functional groups in the 4´-position and were characterized by X-ray crystallography. We aimed to investigate the influence of electron-donating or electron-withdrawing substituents on the inhibitory constant *K*_i_. With the aid of an hCAII crystal structure bound to one of the synthesized azobenzenes, we found that the electronic structure does not strongly affect inhibition. Taken together, all compounds are strong blockers of hCAII with *K*_i_ = 25–65 nM that are potentially photochromic and thus combine studies from chemical synthesis, crystallography and enzyme kinetics.

## Introduction

**Figure 1:**
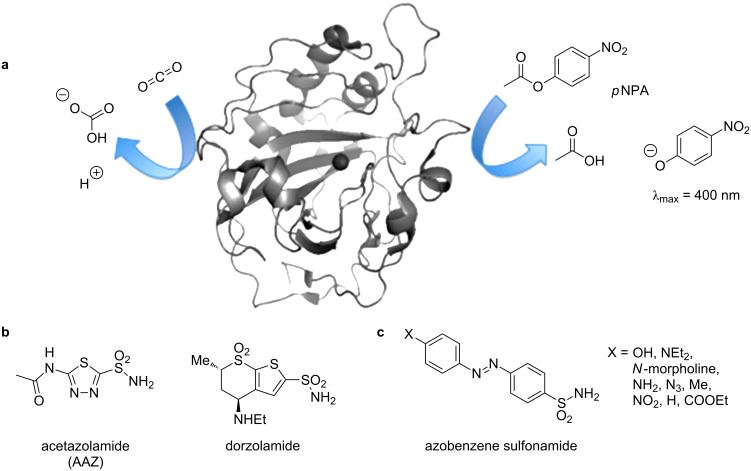
Function and inhibition of hCAII. a) hCAII (pdb: 2vva [7]) catalyzes the hydration of carbon dioxide to bicarbonate and a proton (left) as well as the hydrolysis of *p*NPA to acetate and a colored phenolate (λ_max_ = 400 nm). b) Aryl sulfonamide-containing pharmacophores of hCAII. c) Aryl sulfonamide merged to azobenzenes.

Carbonic anhydrase (CA) is an ubiquitously found zinc-containing metalloenzyme with many isoforms, which all catalyze the conversion of carbon dioxide and water to bicarbonate and a proton (Figure 1a, left) [1]. Despite its native purpose of pH and pressure regulation, its intrinsic esterase activity can be utilized to measure the catalytic activity by hydrolysis of *p*-nitrophenyl actetate (*p*NPA) to a phenolate, of which the product appearance can be observed colorimetrically (Figure 1a, right) [2]. In humans, isoform II (human carbonic anhydrase II; hCAII) is found in many tissues and is responsible for maintaining the inner eye pressure among other regulatory tasks [1]. Consequently, its failure is associated with glaucoma [1,3]. Treatment of this severe disease, that leads to blindness, is achieved with the application of aryl sulfonamides [3]. Being a transition-state analogue [4], this functional group exhibits excellent blocking characteristics of hCAII and culminates its power in many modern marketed drugs, such as acetazolamide (AAZ) or dorzolamide (Figure 1b) [5]. Furthermore, sulfonamide-containing azobenzenes exhibit affinity and blocking ability for hCAII (Figure 1c) [6]. With our knowledge in azobenzene chemistry and photopharmacology, we aimed to further understand how electronic substitution patterns on azobenzenes correlate to changes in enzyme affinity.

## Results and Discussion

**Scheme 1:**
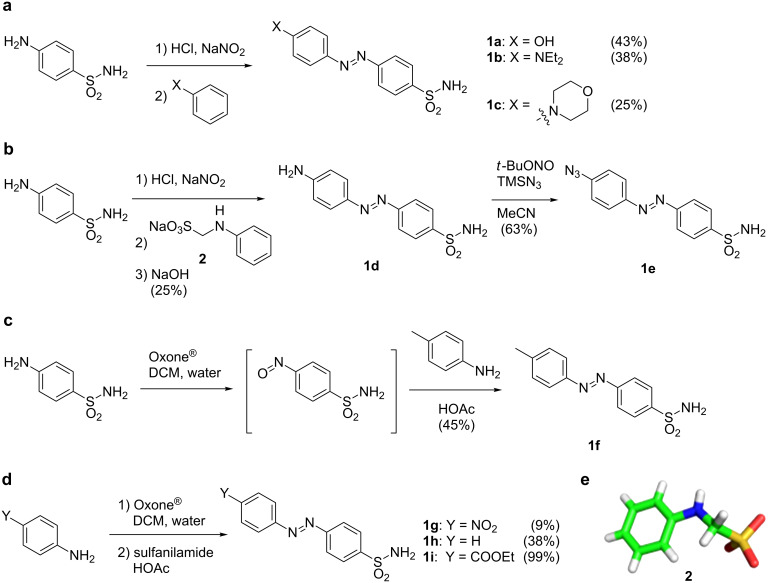
Synthesis and characterization of azobenzene-containing aryl sulfonamides by different strategies. a) Diazotization and trapping of the diazonium salt with an electron-rich aromatic compound yields azobenzenes **1a–c**. b) Reaction of methylene sulfonate-protected aniline and one-pot deprotection yields **1d**, which can be converted to **1e**. c) Mills condensation to obtain **1f**. d) Mills condensation to obtain **1g–i**. e) Crystal structure of sulfonate **2**.

**Figure 2:**
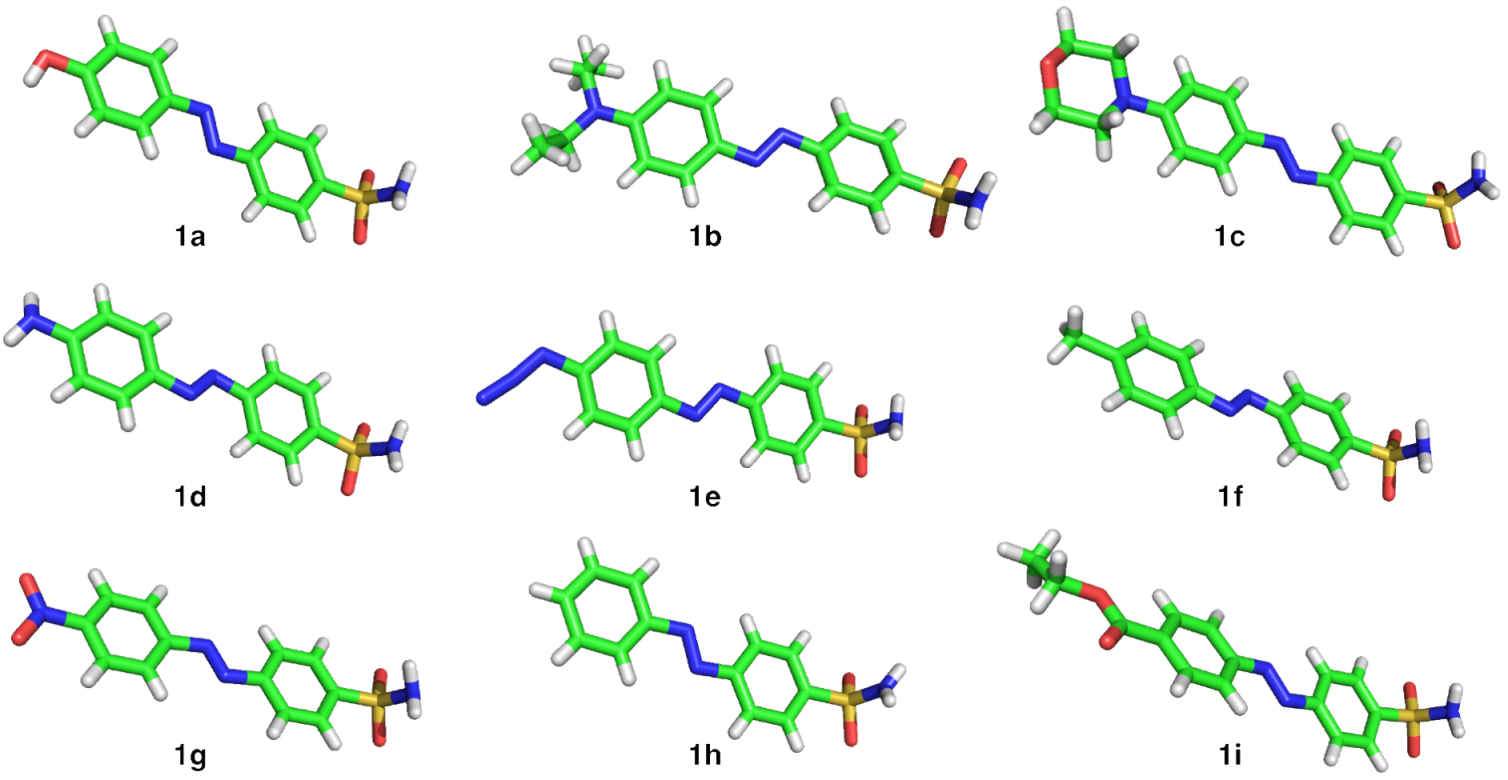
Crystal structures for compounds **1a**–**i** (co-solvents and/or multiple molecules in the asymmetric cell are omitted for clarity).

Azobenzenes can be synthesized by a variety of known chemical transformations [8]. Among them the most widely used is the diazotization of aniline, followed by trapping of the diazonium salt with an electron-rich aromatic compound (such as anilines and phenols). Another commonly used method is the condensation between anilines and aryl nitroso compounds, known as the Mills reaction. According to these transformations, nine sulfonamide containing azobenzenes **1a–i** with different moieties in the 4´-position were synthesized. The substitution in the 4´-position will have the biggest impact on the electronic properties of the sulfonamide group due to communication through the conjugated π-system of the aromatic units and the diazene unit. Commencing with the diazotization of sulfanilamide and subsequent reaction with phenol, *N*,*N*-diethylaniline or *N*-phenylmorpholine led to azobenzenes **1a** [6], **1b** [9] and **1c**, in moderate to low yields (43%, 38% and 25%, respectively) (Scheme 1a). Employing methylene-protected aniline **2** (crystal structure depicted in Scheme 1e) according to the procedure from Supuran and co-workers [6], amino azobenzene **1d** was isolated after a one-pot reaction over three steps in 25%. By diazotization of **1d** and trapping the salt with TMS-azide, we obtained azido azobenzene **1e** in 63% yield through a [3 + 2] and retro-[3 + 2] cycloaddition (Scheme 1b) according to the procedure of Barral et al [10]. For the Mills reaction, different nitroso compounds were generated that were all used without further purification for the following condensation reactions. For example, sulfanilamide reacted with Oxone^®^ in a biphasic DCM/water mixture to its nitroso counterpart, which was condensed with *p*-toluidine to give methyl azobenzene **1f** (Scheme 1c) in 45% yield over two steps. Additionally, several in situ generated nitroso compounds bearing a nitro and a carboxylic acid ester were reacted with sulfanilamide to obtain nitro azobenzene **1g** and ethyl ester azobenzene **1i**, respectively. Commercially available nitrosobenzene gave rise to the unsubstituted sulfonamide **1h** (Scheme 1d). The yield was poor for nitro azobenzene **1g** (9%). Furthermore, the reaction of nitrosobenzene to obtain **1h** proceeded in low yield (38%), while **1i** was isolated in quantitative yield (99%). Crystals suitable for X-ray diffraction were obtained for **2** (Scheme 1e) and all sulfonamide-containing azobenzenes **1a–i** (Figure 2). The crystallization conditions can be found in Supporting Information File 1).

The substitution patterns on the aromatic core together with their electronic characteristics determines the absorption spectra of the individual azobenzenes [11]. We assessed the π–π*-band wavelength of maximal absorption (λ_max_) by RP–LCMS equipped with a UV–vis diode array detector, when determining the purity of our library. Therefore, λ_max_ is determined in a water/acetonitrile mixture, which mimics aqueous conditions that are also used for the biological assays. The results are given in Table 1. While the “naked” azobenzene **1h** has the absorbance maximum in the bluest part of the spectrum (λ_max_ (**1h**) = 322 nm), substitution on the azobenzene in the 4´-position leads to a bathochromic shift, which is smaller for electron-withdrawing groups (λ_max_ (**1g**) = 328 nm; λ_max_ (**1i**) = 342 nm) and more pronounced for electron-donating groups (λ_max_ (**1f**) = 338 nm, λ_max_ (**1a**) = 358 nm, λ_max_ (**1d**) = 404 nm, λ_max_ (**1c**) = 414 nm, and λ_max_ (**1b**) = 460 nm). Interestingly, azide **1e** with a Hammett constant of σ = 0.08 [12], exhibits a bathochromic shift (λ_max_ (**1e**) = 356 nm) close to hydroxy azobenzene **1a** although it can be considered neither electron-withdrawing, nor electron-donating.

**Table 1:** Maximal absorbance wavelength (λ_max_), Hammett constants (σ) and inhibitory characteristics (IC_50_ and *K*_i_) of **1a**–**i**.

	4´-substitution pattern	λ_max_ (nm)	Hammett constant σ [12]	IC_50_ (nM)	*K*_i_ (nM)

**1a**	OH	358	−0.37	165.6	29.7
**1b**	NEt_2_	460	−0.83	139.6	25.0
**1c**	*N*-morpholine	414	−0.83^a^	309.2	55.4
**1d**	NH_2_	404	−0.66	171.4	30.7
**1e**	N_3_	356	+0.08	257.1	46.1
**1f**	Me	338	−0.17	363.2	65.1
**1g**	NO_2_	328	+0.78	159.4	28.6
**1h**	H	322	+0.00	249.7	44.8
**1i**	COOEt	342	+0.45	167.9	30.1
AAZ	–	–	–	55.5	10.0

^a^To the best of our knowledge the Hammett constant for morpholine has not been previously determined, therefore we used the parameter for alkylated amines due to its similar electronic nature.

**Figure 3:**
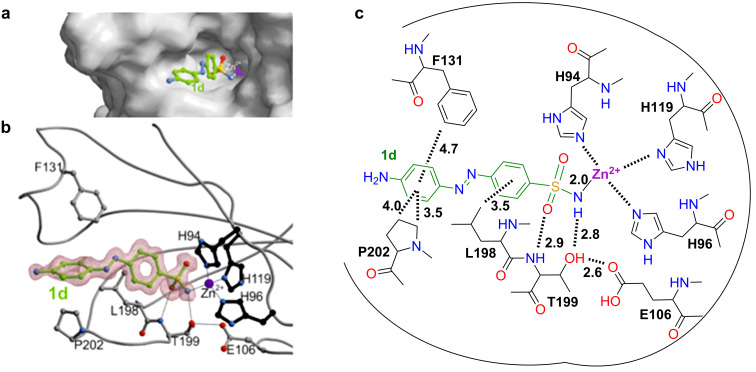
Crystal structure of hCAII bound to **1d** (pdb: 5byi). a) The terminal amine of **1d** is solvent-exposed, while the azobenzene is sticking in the cavity. b) Electron-density map of **1d** bound to zinc with primary interactions. c) Interactions of **1d** in the catalytic site in angstroms (Å). Primary interactions of the zinc-bound sulfonamide can be seen to T199 and L198 to the aromatic core. Secondary interactions can be observed from F131 and P202 towards the second aromatic ring of the azobenzene.

To gain a deeper understanding into the binding mode of the synthesized azobenzene-containing sulfonamides we set out to co-crystallize an inhibitor with the wild-type enzyme. Protein crystals bound to **1d** (Figure 3a, pdb: 5byi) were obtained using a previously reported method [13]. Due to the rigidness of the azobenzene the sulfonamide nitrogen and the 4´-position are far apart (>12 Å). The moiety in this position is solvent exposed and should therefore not contribute to the binding affinities by direct interactions (Figure 3a and b). Apart from primary binding interactions between the sulfonamide to the zinc center and T199, both of which are well-described [1] (Figure 3b and c), we were looking for secondary interactions resulting from the azobenzene (Figure 3c). Indeed, we found that the methyl group of L198 interacts with the sulfonamide-bearing aromatic core with a carbon to centroid distance of 3.5 Å. Furthermore, P202 and F131 contribute to centroid interactions with the second aromatic ring with distances of 3.5 (N*C*H_2_ to centroid), 4.0 (NCH_2_*C*H_2_ to centroid) and 4.7 Å (*C*H to centroid), respectively. These interactions can be weaker or stronger depending on the functional group in the 4´-position, as they affect the electronic properties of the aromatic system, and this would also be reflected by the Hammett constant. Interestingly, the azobenzene does not adopt a completely planar shape but is distorted with dihedral angles of −143.3° and 23.8° at the N=N bond and at the second aromatic ring to the diazene unit, respectively. It should be noted that a water molecule is held by the peptide backbone in the gorge (2.6 Å to *O*H of T200 and 2.7 Å to C*O* of P202), which might have interactions with the diazene unit although the distance for classical hydrogen bonding is rather long (3.2 Å).

In order to determine the half-maximal inhibitory concentrations (IC_50_) and the inhibitory constants (*K*_i_) towards hCAII for our library, we used a colorimetric endpoint measurement of the catalyzed *p*NPA hydrolysis (Figure 1a). Usually, a dansyl competition assay is employed for this purpose [1,14]. However, as this assay is fluorescence-based and azobenzenes can quench fluorescence [15], this might cause a distortion in the obtained data. Furthermore, irradiation with UV light (i.e., λ = 280 nm for tryptophan excitation) can result in azobenzene-*cis*-isomerization, which could lead to different binding characteristics. Therefore, we aimed at the endpoint absorbance system described herein. After expression and purification of wild-type hCAII we tested the benchmark blocker AAZ (Figure 1b) and obtained a *K*_i_ = 10.0 nM, which is in accordance with a previously reported inhibition constant (*K*_i_ (AAZ) = 12 nM [16]). Consequently, we were confident that our assay could assess the inhibitory characteristics of our library in a robust, reliable and reproducible manner.

**Figure 4:**
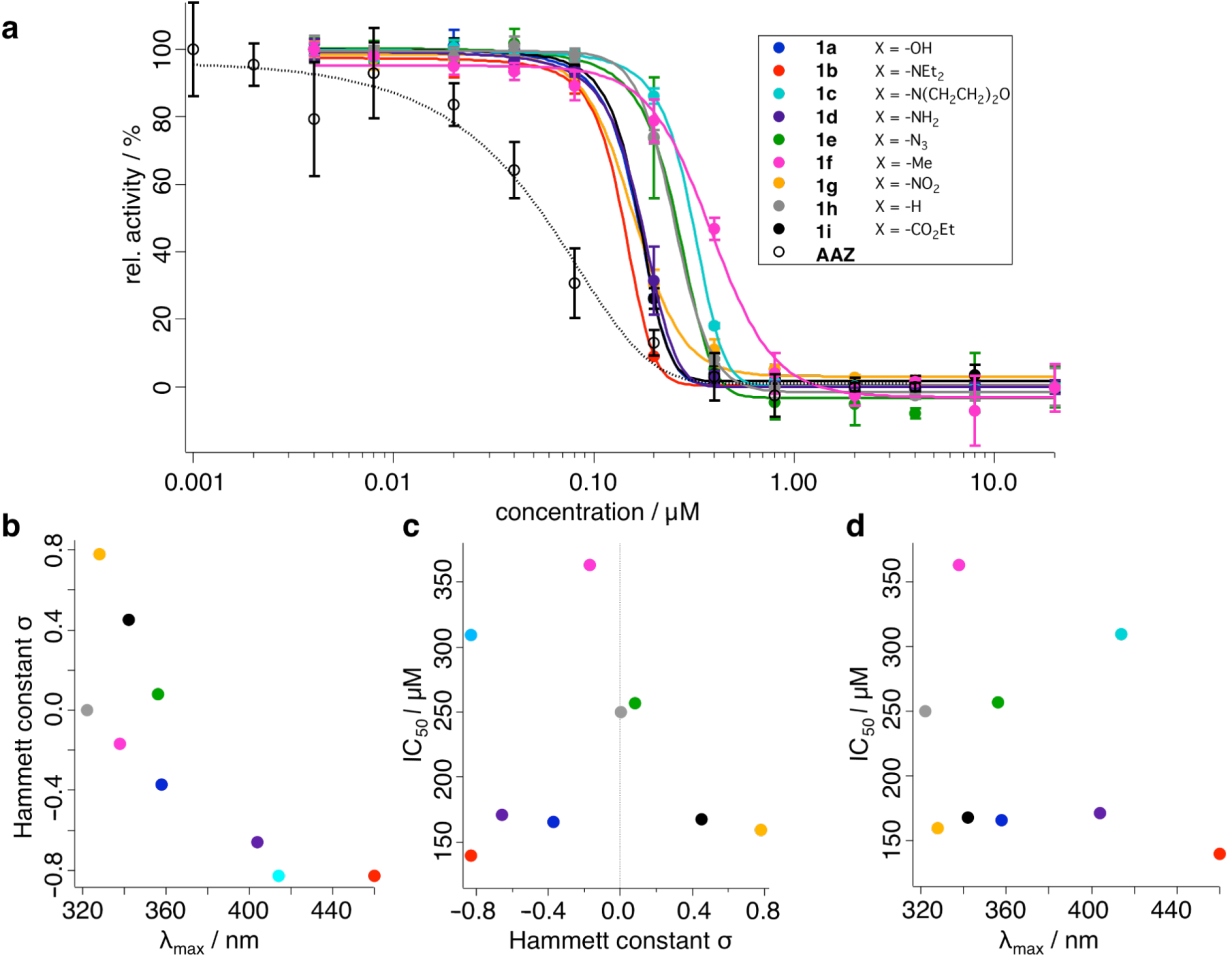
Inhibition of hCAII by electronically different azobenzene sulfonamides and AAZ. a) Endpoint measurement for the determination of IC_50_ for compounds **1a**–**i**. b) Hammett constants versus maximal absorbance wavelength shows decreasing trend. c) IC_50_ versus Hammett constants. d) IC_50_ versus λ_max_.

By using the Cheng–Prusoff equation [17] with a Michaelis–Menten constant of *K*_m_ = 1092.5 µM for *p*NPA (see Supporting Information File 1, Figure S2), we calculated the inhibitory constant *K*_i_ (Table 1) for each compound from the IC_50_ values obtained from sigmoidal fitting of the activity vs. concentration curve (see Figure 4a and Supporting Information File 1 for details). Azobenzenes **1a** and **1d** have been synthesized and tested previously (with a CO_2_ hydration assay), and the *K*_i_ values determined in the previous work are one order of magnitude higher than in our findings (*K*_i_ (**1a**) = 665 nM or 29.7; *K*_i_ (**1d**) = 106 nM or 30.7) towards hCAII [6]. Interestingly, in our studies the most efficient blocker turned out to be **1b** with a *K*_i_ = 25.0 nM, which also shows the greatest red-shift in its maximal absorbance wavelength (π–π* band). Another electron-donating blocker bearing a methyl group substituent (**1f**), however, had the lowest affinity (*K*_i_ = 65.1 nM) of the library. Compound **1c** offers the second-lowest affinity (*K*_i_ = 55.4 nM), which seems counter-intuitive, as the only difference with respect to **1b** is the connection of the ethyl chains by an oxygen atom to a morpholine ring. This does not only affect the binding properties, but also the π–π* band, which is 46 nm blue-shifted relative to **1b**. The inhibitors with the proton and azide substituents (**1h** and **1e**, respectively) show very similar affinities towards hCAII with *K*_i_ = 44.8 nM and *K*_i_ = 46.1 nM. Sterics are also restrained, but should not affect binding, as the 4´-position is solvent-exposed (vide supra). Taking all of these findings into account, we conclude that the sulfonamide–zinc interaction dominates the binding affinity.

The electronic differences of our azobenzene library is expressed by their absorption spectra (as an indicator for the electron richness of the azobenzene) or in their Hammett constants (as an indicator for electron-pushing or pulling effects). When plotted against each other a trend can be observed, which is reflected by a more bathochromic shift when the Hammett constant becomes more negative (Figure 4b). However, when plotting the Hammett constants or the maximal absorbance wavelength versus the IC_50_ (Figure 4c and d, respectively), no clear correlation can be found. In both cases morpholine **1c**, azide **1e**, methyl **1f** and unsubstituted azobenzene sulfonamide **1h** lie in the same region (50 µM), while all other inhibitors (hydroxy **1a**, alkyl amine **1b**, amine **1d**, nitro **1h** and ethyl carboxylate **1i**) show higher affinities at around 30 µM. The latter also shows a distribution from UV to blue maximal absorbance. We therefore speculate that electronics do not have a primary effect on the binding properties towards hCAII, but rather the sole presence of an aryl sulfonamide is sufficient. It should be pointed out that “naked” azobenzene **1h** does not follow the trend when correlating its Hammett constant of σ = 0.00 to its λ_max_ = 322 nm. As the plots from Figure 4c and d can be considered mirror images of each other, compound **1h** does not fit into this picture.

## Conclusion

In conclusion, we have synthesized a small library of nine azobenzene sulfonamides with differing substitution patterns in the 4´-position using either azo coupling reactions or the Mills reaction. We determined the π–π* band and the crystal structures of all nine compounds together with the protein crystal structure of hCAII bound to inhibitor **1d**. The latter structure highlights the interactions of the sulfonamide and the azobenzene with the protein cavity. The inhibitory action on hCAII was tested for all compounds using an endpoint measurement of catalytic *p*NPA hydrolysis. The inhibitory constants were in close proximity to each other (*K*_i_ = 25–65 nM) and a correlation of electron density as characterized by the Hammett constant σ with the binding affinities was not observed. We have expanded the repertoire of sulfonamide blockers of hCAII and described synthetic routes to potentially photochromic representatives. Furthermore, the protein crystal structure of hCAII bound to **1d** described herein can be used as a template for the rational design of novel hCAII blockers. The biological activity of these blockers is currently under investigation and the results will be published in due course.

## Supporting Information

File 1 Chemical procedures, spectral data and X-ray crystallographic tables. Protein purification, crystallization conditions and measurement of Michaelis–Menten constant.
