# Supplementary material for: Azobenzene-based inhibitors of human carbonic anhydrase II
Source: Beilstein J Org Chem. 2015 Jul 7;11:1129–35. doi: 10.3762/bjoc.11.127 (PMC4505086; doi:10.3762/bjoc.11.127)
Supplement: File 1 — Chemical procedures, spectral data and X-ray crystallographic tables. Protein purification, crystallization conditions and measurement of Michaelis–Menten constant. [file Beilstein_J_Org_Chem-11-1129-s001.pdf]

Supporting Information

for

Azobenzene-based inhibitors of human carbonic anhydrase II

Leander Simon Runtsch[1], David Michael Barber[1], Peter Mayer[1], Michael Groll[2], Dirk Trauner[1,]* and Johannes Broichhagen[1,]*

Address: [1]Department of Chemistry, Ludwig-Maximilians-University Munich and Munich Center for Integrated Protein Science, Butenandtstrasse 5–13, 81377 Munich, Germany and [2]Department of Biochemistry, Technical University Munich and Munich Center for Integrated Protein Science, Lichtenbergstr. 4, 85748 Garching, Germany

Email: Dr. Johannes Broichhagen - johannes.broichhagen@cup.lmu.de; Prof. Dr. Dirk Trauner - dirk.trauner@lmu.de

*Corresponding author

Chemical procedures, spectral data and X-ray crystallographic tables. Protein purification, crystallization conditions and measurement of Michaelis–Menten constant

Table of Contents

1. **General Experimental** S4

2. **Synthesis** S6
   - 2.1. **Mills reaction procedure (A) for azobenzene formation** S6
   - 2.2. **Diazonium coupling procedure (B) for azobenzene formation** S6
   - 2.3. **Aniline oxidation procedure (C) for nitroso formation** S6

2.4. 4-Nitrosobenzenesulfonamide S7
2.5. Ethyl 4-nitrosobenzoate S7
2.6. 1-Nitro-4-nitrosobenzene S7
2.7. (*E*)-4-(4-Hydroxyphenyldiazenyl)benzenesulfonamide (1a) S8
2.8. (*E*)-4-((4-(Diethylamino)phenyl)diazenyl)benzenesulfonamide (1b) S9
2.9. (*E*)-4-((4-Morpholinophenyl)diazenyl)benzenesulfonamide (1c) S10
2.10. (*E*)-4-(4-Aminophenyldiazenyl)benzenesulfonamide (1d) S11
2.11. (*E*)-4-(4-Azidophenyldiazenyl)benzenesulfonamide (1e) S12
2.12. (*E*)-4-(*p*-Tolyldiazenyl)benzenesulfonamide (1f) S13
2.13. (*E*)-4-((4-Nitrophenyl)diazenyl)benzenesulfonamide (1g) S14
2.14. (*E*)-4-(Phenyldiazenyl)benzenesulfonamide (1h) S15
2.15. (*E*)-Ethyl 4-((4-sulfamoylphenyl)diazenyl)benzoate (1i) S16
2.16. Sodium (phenylamino)methanesulfonate hydrate (2) S17

3. Spectra S18
3.1. (*E*)-4-(4-Hydroxyphenyldiazenyl)benzenesulfonamide (1a) S18
3.2. (*E*)-4-((4-(Diethylamino)phenyl)diazenyl)benzenesulfonamide (1b) S19
3.3. (*E*)-4-((4-Morpholinophenyl)diazenyl)benzenesulfonamide (1c) S20
3.4. (*E*)-4-(4-Aminophenyldiazenyl)benzenesulfonamide (1d) S21
3.5. (*E*)-4-(4-Azidophenyldiazenyl)benzenesulfonamide (1e) S22
3.6. (*E*)-4-(*p*-Tolyldiazenyl)benzenesulfonamide (1f) S23
3.7. (*E*)-4-((4-Nitrophenyl)diazenyl)benzenesulfonamide (1g) S24
3.8. (*E*)-4-(Phenyldiazenyl)benzenesulfonamide (1h) S25
3.9. (*E*)-Ethyl 4-((4-sulfamoylphenyl)diazenyl)benzoate (1i) S26
3.10. Sodium (phenylamino)methanesulfonate hydrate (2) S27

4. X-Ray crystallographic data S28
4.1. (*E*)-4-((4-(Hydroxyphenyl)diazenyl)benzenesulfonamide (1a) S29
4.2. (*E*)-4-((4-(Diethylamino)phenyl)diazenyl)benzenesulfonamide (1b) S31
4.3. (*E*)-4-((4-Morpholinophenyl)diazenyl)benzenesulfonamide (1c) S33
4.4. (*E*)-4-(4-Aminophenyldiazenyl)benzenesulfonamide (1d) S35
4.5. (*E*)-4-(4-Azidophenyldiazenyl)benzenesulfonamide (1e) S37
4.6. (*E*)-4-(*p*-Tolyldiazenyl)benzenesulfonamide (1f) S39
4.7. (*E*)-4-((4-Nitrophenyl)diazenyl)benzenesulfonamide (1g) S41
4.8. (*E*)-4-((Phenyl)diazenyl)benzenesulfonamide (1h) S43

4.9. (*E*)-Ethyl 4-((4-sulfamoylphenyl)diazenyl)benzoate (1i) S45
4.10. Sodium (phenylamino)methanesulfonate hydrate (2) S47
4.11. wt hCAII bound to 1d S49
5. hCAII purification, crystallization and assay S51
5.1. Purification and crystallization S51
5.2. Determination of half-maximal inhibitory concentration (IC_{50}) S51
5.3. Determination of inhibitory constants (K_i) S52
6. Supplementary Figures S53
7. References S54

1. *General Experimental*

Solvents for chromatography and reactions were purchased in HPLC grade or distilled from an appropriate drying reagent prior to use. If necessary, solvents were degassed either by freeze-pump-thaw or by bubbling N_2 through the vigorously stirred solution for several minutes. Unless otherwise stated, all other reagents were used without further purification from commercial sources.

Flash column chromatography was carried out on silica gel 60 (0.040–0.063 mm) purchased from Merck. Reactions and chromatography fractions were monitored by thin layer chromatography (TLC) on Merck silica gel 60 F254 glass plates. The plates were visualized under UV light at 254 nm or with an appropriate staining method (iodine, *p*-anisaldehyde, $KMnO_4$) followed by heating.

NMR spectra were recorded in deuterated solvents on VARIAN Mercury 200, BRUKER AXR 300, VARIAN VXR 400 S, BRUKER AMX 600 and BRUKER Avance III HD 400 (equipped with a CryoProbe™) instruments and calibrated to residual solvent peaks ($^1H/^{13}C$ in ppm): $CDCl_3$ (7.26/77.16), DMSO-d_6 (2.50/39.52), MeCN-d_3 (1.94/1.32), acetone-d_6 (2.05/29.84), CD_3OD (3.31/49.00). Multiplicities are abbreviated as follows: s = singlet, d = doublet, t = triplet, q = quartet, br = broad, m = multiplet. Spectra are reported based on appearance, not on theoretical multiplicities derived from structural information.

A Varian MAT CH7A mass spectrometer was used to obtain low- and high resolution electron impact (EI) mass spectra. Low- and high resolution electrospray (ESI) mass spectra were obtained on a Varian MAT 711 MS instrument operating in either positive or negative ionization modes.

UV–vis spectra were recorded on a Varian Cary 50 Bio UV–Vis Spectrophotometer using Helma SUPRASIL precision cuvettes (10 mm light path) equipped with a Polychrome V (Till Photonics) monochromator.

LC–MS was performed on an Agilent 1260 Infinity HPLC System, MS-Agilent 1100 Series, Type: 1946D, Model: SL, equipped with a Agilent Zorbax Eclipse Plus C18 (100

x 4.6 mm, particle size 3.5 micron) RP column with a constant flow rate of 2 mL/min. Retention times (t_R) are given in minutes (min).

IC_{50} values were measured on a BMG LABTECH's Omega Series FLUOstar microplate reader with clear flat-bottom white 96-well plates (Greiner Bio-One).

IR spectra were recorded on a Perkin Elmer Spectrum BX II FTIR system. The measured wave numbers are reported in cm^{-1}.

Melting points were measured on the apparatus Büchi Melting Point B-540 from *BÜCHI Labortechnik AG* or on an EZ-Melt apparatus form *Stanford Research Systems* and are uncorrected.

Single crystal X-ray diffraction experiments were performed on a Bruker TXS diffractometer equipped with a multilayer monochromator, a Photon 100 detector, and a rotating-anode generator (Mo Kα radiation). The data of **2** have been collected on an Oxford Diffraction Xcalibur diffractometer equipped with a Mo sealed-tube source (Mo Kα radiation). The data have been collected at 173 K with the exception of **1b**, **1c** and **1h** (100 K). The SADABS program embedded in the Bruker APEX2 software has been used for absorption corrections in all structures but **2** (ABSPACK program embedded in the CrysAlisPro sofrware package).

2. *Synthesis*

2.1. Mills reaction procedure (A) for azobenzene formation

A round bottomed flask was charged with aniline (1.0 equiv) dissolved in HOAc (and if necessary an organic co-solvent). To this solution nitrosobenzene (1.2–3.0 equiv) was added in one portion and the mixture was stirred at rt or 60 °C overnight. Then it was neutralized by the addition of aqueous sat. $NaHCO_3$ and extracted with EtOAc (3x). The combined organic layers were washed with sat. $NaHCO_3$ and brine before they were dried over $MgSO_4$ and subjected to flash column chromatography.

2.2. Diazonium coupling procedure (B) for azobenzene formation

As described and reported in ref [1], a round bottomed flask was charged with aniline (1.0 equiv) dissolved in 2.4 M HCl and cooled to 0 °C. Aqueous $NaNO_2$ (1.2 equiv, 2.3 M) was added dropwise to form the diazonium salt, which resulted in a yellow color. The mixture was stirred for 5–10 min at 0 °C before it was transferred to a solution of the corresponding coupling partner in aqueous 1 M NaOAc (with the addition of methanol until everything was dissolved). The solution turned red immediately and was allowed to warm to rt. Workup procedure depended on the azobenzene formed and can be found at the appropriate compounds.

2.3. Aniline oxidation procedure (C) for nitroso formation

A round bottomed flask was charged with aniline (1.0 equiv) in DCM and water was added. Oxone® (2.0 equiv) was added and the biphasic system was stirred vigorously for 3 h. The layers were separated and the green organic layer was washed with 1 M HCl (2x), water and brine before it was dried over $MgSO_4$. After removal of all volatiles, the crude nitroso compound was isolated as a solid, which was used without further purification.

2.4. 4-Nitrosobenzenesulfonamide

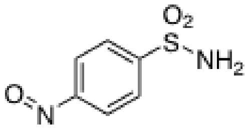

4-Nitrosobenzenesulfonamide was synthesized via procedure C.

Amounts: sulfanilamide (4.00 g, 23.2 mmol), Oxone® (14.2 g, 46.4 mmol), DCM/water 75:150 mL.

2.5. Ethyl 4-nitrosobenzoate

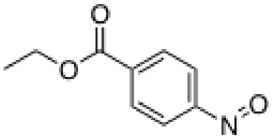

Ethyl 4-nitrosobenzoate was synthesized via procedure C.

Amounts: 4-amino ethylbenzoate (4.00 g, 24.2 mmol), Oxone® (14.9 g, 48.4 mmol), DCM/water 75:150 mL.

2.6. 1-Nitro-4-nitrosobenzene

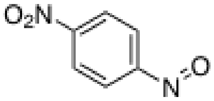

1-Nitro-4-nitrosobenzene was synthesized via procedure C.

Amounts: 4-nitroaniline (6.00 g, 46.8 mmol), Oxone® (28.8 g, 93.6 mmol), DCM/water 150:150 mL.

2.7. (*E*)-4-(4-Hydroxyphenyldiazenyl)benzenesulfonamide (1a)

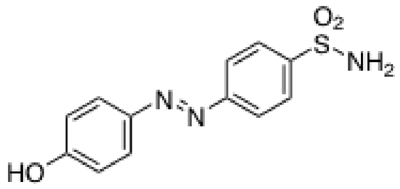

(*E*)-4-(4-hydroxyphenyldiazenyl)benzenesulfonamide [2] was prepared according to procedure B. After completion of the reaction, the product was precipitated by the addition of 2 M HCl and extracted with EtOAc. The organic layer was washed with brine and dried over $MgSO_4$. After removal of all volatiles, the solid was recrystallized from hot acetone overnight to obtain 3.47 g (12.5 mmol, 43%) of the desired product as a red solid.

Amounts: sulfanilamide (1.0 equiv, 5.00 g, 29.0 mmol), phenol (1.0 equiv, 2.73 g, 29.0 mmol), $NaNO_2$ (1.2 equiv, 2.4 g, 34.8 mmol).

^{1}H NMR (400 MHz, DMSO-d_6): δ [ppm] = 10.48 (br s, 1H), 8.01 (d, J = 8.6 Hz, 2H), 7.96 (d, J = 8.7 Hz, 2H), 7.86 (d, J = 8.8 Hz, 2H), 7.51 (br s, 2H), 6.98 (d, J = 8.9 Hz, 2H).

^{13}C NMR (101 MHz, DMSO-d_6): δ [ppm] = 161.8, 153.7, 145.3, 145.0, 127.0, 125.4, 122.5, 116.2.

HRMS (ESI): calc. for $C_{12}H_{10}N_3O_3S^-$ (M-H)$^-$: 276.0448, found: 276.0447.

UV/Vis (LCMS): λ_{max} = 358 nm.

t_R (LCMS; MeCN/H_2O/formic acid = 10/90/0.1 → 90/10/0.1 over 7 min) = 3.067 min.

IR (ATR): wave number/cm^{-1} = 3345, 3241, 1595, 1502, 1427, 1300, 1266, 1156, 1138, 1089, 901, 848, 796, 709.

m.p. = decomposition >250 °C

2.8. (*E*)-4-((4-(Diethylamino)phenyl)diazenyl)benzenesulfonamide (1b)

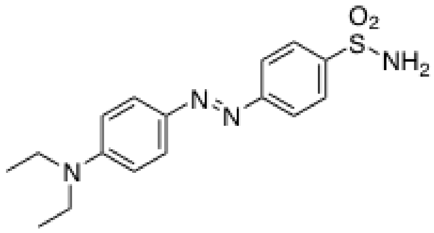

(*E*)-4-((4-(Diethylamino)phenyl)diazenyl)benzenesulfonamide was prepared according to procedure A as reported before and spectra matched the one reported [1]. Flash column chromatography (25% EtOAc/isohexanes) was performed to obtain 1.45 g (4.37 mmol, 38%) of the desired product as a red solid.

Amounts: sulfanilamide (1.0 equiv, 2.00 g, 11.6 mmol), $NaNO_2$ (1.2 equiv, 0.96 g, 13.9 mmol), *N*,*N*-diethylaniline (1.0 equiv, 1.73 g, 11.6 mmol, 1.84 mL).

^{1}H NMR (400 MHz, DMSO-d_6): δ [ppm] = 7.95 (d, J = 8.6 Hz, 2H), 7.88 (d, J = 8.6 Hz, 2H), 7.81 (d, J = 9.2 Hz, 2H), 7.45 (s, 2H), 6.82 (d, J = 9.3 Hz, 2H), 3.47 (q, J = 7.0 Hz, 4H), 1.15 (t, J = 7.0 Hz, 6H).

^{13}C NMR (101 MHz, DMSO-d_6): δ [ppm] = 154.2, 150.8, 143.8, 142.2, 126.9, 125.8, 121.9, 111.1, 44.2, 12.5.

HRMS (ESI): calc. for $C_{16}H_{21}N_4O_2S^+$ $(M+H)^+$: 333.1380, found: 333.1377.

UV/Vis (LCMS): λ_{max} = 460 nm.

t_R (LCMS; MeCN/H_2O/formic acid = 10/90/0.1 → 90/10/0.1 over 7 min) = 4.364 min.

IR (ATR): wave number/cm^{-1} = 3364, 3261, 2978, 1603, 1585, 1513, 1404, 1386, 1353, 1331, 1313, 1302, 1277, 1195, 1164, 1138, 1102, 1083, 1015, 884, 848, 821, 738, 681.

m.p. = 190-195 °C

2.9. (*E*)-4-((4-Morpholinophenyl)diazenyl)benzenesulfonamide (1c)

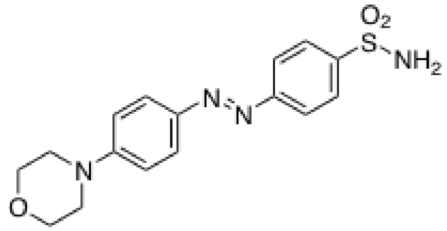

(*E*)-4-((4-Morpholinophenyl)diazenyl)benzenesulfonamide was prepared according to procedure B. After completion of the reaction, the resulting solid was filtered off and washed twice with water and finally recrystallized from acetone to obtain 991 mg (2.86 mmol, 25%) of the desired product as a red solid.

Amounts: sulfanilamide (1.0 equiv, 2.00 g, 11.6 mmol), *N*-phenyl morpholine (1.0 equiv, 1.89 g, 11.6 mmol), $NaNO_2$ (1.2 equiv, 0.96 g, 13.9 mmol).

^{1}H NMR (400 MHz, DMSO-d$_6$): δ [ppm] = 7.98 (d, J = 8.7 Hz, 2H), 7.94 (d, J = 8.7 Hz, 2H), 7.85 (d, J = 9.1 Hz, 2H), 7.49 (br s, 2H), 7.10 (d, J = 9.2 Hz, 2H), 3.75 (m, 4H), 3.35 (m, 4H).

^{13}C NMR (101 MHz, DMSO-d$_6$): δ [ppm] = 153.9, 153.6, 144.6, 144.1, 127.0, 125.0, 122.3, 113.8, 65.9, 46.9.

HRMS (ESI): calc. for $C_{16}H_{17}N_4O_3S^-$ (M-H)$^-$: 345.1027, found: 345.1026.

UV/Vis (LCMS): λ_{max} = 414 nm.

tt_R (LCMS; MeCN/H_2O/formic acid = 10/90/0.1 → 90/10/0.1 over 7 min) = 3.612 min.

IR (ATR): wave number/cm^{-1} = 3197, 3081, 2859, 1600, 1506, 1448, 1380, 1336, 1301, 1269, 1234, 1160, 1141, 1110, 1089, 1068, 1051, 917, 852, 826, 740, 698.

m.p. = decomposition 220 °C

2.10. (*E*)-4-(4-Aminophenyldiazenyl)benzenesulfonamide (1d)

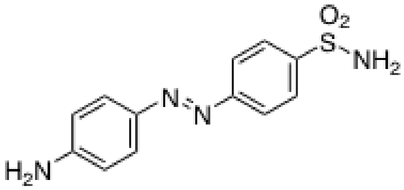

(*E*)-4-(4-Aminophenyldiazenyl)benzenesulfonamide was prepared according to a published procedure [2].

^{1}H NMR (400 MHz, DMSO-d$_6$): δ [ppm] = 7.94 (d, *J* = 8.6 Hz, 2H), 7.87 (d, *J* = 8.6 Hz, 2H), 7.71 (d, *J* = 8.8 Hz, 2H), 7.45 (s, 2H), 6.69 (d, *J* = 8.8 Hz, 2H), 6.33 (br s, 2H).

^{13}C NMR (101 MHz, DMSO-d$_6$): δ [ppm] = 154.2, 153.8, 143.8, 142.8, 126.9, 125.9, 122.0, 113.5.

HRMS (ESI): calc. for $C_{12}H_{13}N_4O_2S^+$ $(M+H)^+$: 277.0754, found: 277.0753.

UV/Vis (LCMS): λ (π → π*) = 404 nm.

t$_R$ (LCMS; MeCN/H_2O/formic acid = 10/90/0.1 → 90/10/0.1 over 7 min) = 3.000 min.

IR (ATR): wave number/cm^{-1} = 3378, 3347, 3256, 3046, 1620, 1600, 1503, 1424, 1394, 1330, 1300, 1235, 1162, 1137, 1089, 1010, 901, 849, 839, 706.

m.p. = 240 °C

2.11. (*E*)-4-(4-Azidophenyldiazenyl)benzenesulfonamide (1e)

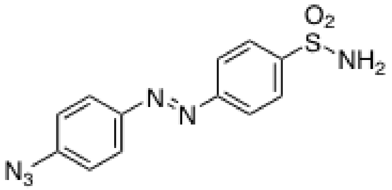

A solution of 126 mg (1.0 equiv, 0.46 mmol) 4-((4-aminophenyl)-diazenyl)benzenesulfonamide (**1d**) in 20 mL MeCN was cooled to −10 °C and 72 mg (1.5 equiv, 0.70 mmol, 82 µL) of *t*-BuONO was added, followed by 10 drops of TFA. The color of the reaction mixture turned to deep red. After stirring for 5 min, 64 mg (1.2 equiv, 0.56 mmol, 74 µL) of $TMSN_3$ was added dropwise to the solution and it was stirred for another hour after which it was allowed to warm to rt. The reaction mixture was diluted with 25 mL of H_2O and extracted with EtOAc. The organic layer was separated, washed with H_2O (3 x 50 mL) and brine and finally dried over $MgSO_4$. Flash column chromatography (50% EtOAc/pentane) was performed to obtain 88.0 g (0.29 mmol, 63%) of the desired product as a red solid.

^{1}H NMR (400 MHz, MeCN-d_3) δ [ppm] = 8.06–7.98 (m, 6H), 7.27 (d, *J* = 8.9 Hz, 2H), 5.79 (br s, 2H).

^{13}C NMR (101 MHz, MeCN-d_3): δ [ppm] = 154.3, 149.5, 144.8, 143.9, 127.3, 124.8, 123.0, 120.0.

HRMS (ESI): calc. for $C_{12}H_9N_6O_2S^-$ $(M\text{-}H)^-$: 301.0513, found: 301.0519.

UV/Vis (LCMS): λ ($\pi \rightarrow \pi^*$) = 356 nm; λ ($n \rightarrow \pi^*$) = 443 nm.

t_R (LCMS; MeCN/H_2O/formic acid = 10/90/0.1 → 90/10/0.1 over 7 min) = 4.190 min.

IR (ATR): wave number/cm^{-1} = 3365, 3266, 2117, 1595, 1496, 1335, 1302, 1276, 1162, 1144, 1126, 1104, 1092, 1012, 894, 849, 718, 661.

m.p. = decomposition >175 °C

2.12. (*E*)-4-(*p*-Tolyldiazenyl)benzenesulfonamide (1f)

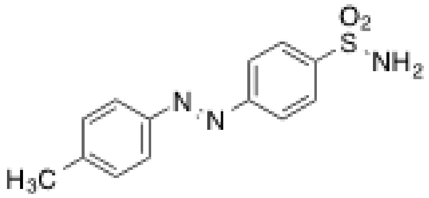

(*E*)-4-(*p*-Tolyldiazenyl)benzenesulfonamide was prepared according to procedure A. Flash column chromatography (100% DCM) was performed to obtain 22.6 g (0.08 mmol, 45%) of the desired product as a red solid.

Amounts: nitroso sulfanilamide (3.0 equiv, 100.9 mg, 0.54 mmol), *p*-toluidine (1.0 equiv, 19.5 mg, 0.18 mmol), HOAc (10 mL).

^{1}H NMR (400 MHz, DMSO-d_6): δ [ppm] = 8.02 (s, 4H), 7.85 (d, J = 8.3 Hz, 2H), 7.55 (s, 2H), 7.47–7.40 (d, J = 8.3 Hz, 2H), 2.42 (s, 3H).

^{13}C NMR (101 MHz, DMSO-d_6): δ [ppm] = 153.4, 150.0, 145.8, 142.8, 130.1, 127.1, 123.0, 122.9, 21.2.

HRMS (ESI): calc. for $C_{13}H_{14}N_3O_2S^+$ $(M+H)^+$: 276.0801, found: 276.0799.

UV/Vis (LCMS): λ_{max} ($\pi \rightarrow \pi^*$) = 334 nm, λ_{max} ($n \rightarrow \pi^*$) = 448 nm.

t_R (LCMS; MeCN/H_2O/formic acid = 10/90/0.1 → 90/10/0.1 over 7 min) = 3.355 min (*cis*); 4.133 min (*trans*).

IR (ATR): wave number/cm^{-1} = 3355, 3261, 1602, 1548, 1500, 1452, 1399, 1382, 1342, 1302, 1226, 1175, 1162, 1144, 1105, 1090, 1012, 905, 851, 826, 782, 749, 726, 704.

m.p. = 215-220 °C

2.13. (*E*)-4-((4-Nitrophenyl)diazenyl)benzenesulfonamide (1g)

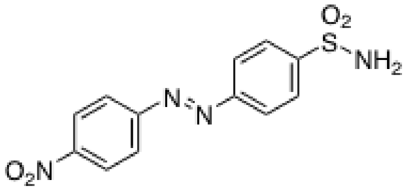

(*E*)-4-((4-Nitrophenyl)diazenyl)benzenesulfonamide was prepared according to procedure A. Flash column chromatography (acetone/isohexanes = 3:7 → 1:1) was performed to obtain 448 mg (1.46 mmol, 9%) of the desired product as a red solid.

Amounts: sulfanilamide (1.0 equiv, 2.80 g, 16.3 mmol), 1-nitro-4-nitrosobenzene (1.0 equiv, 2.48 g, 16.3 mmol), HOAc/DCM 1:1 (40 mL).

^{1}H NMR (400 MHz, DMSO-d_6): δ [ppm] = 8.47 (d, J = 9.0 Hz, 2H), 8.17–8.11 (m, 4H), 8.07 (d, J = 8.6 Hz, 2H), 7.60 (br s, 2H).

^{13}C NMR (101 MHz, DMSO-d_6): δ [ppm] = 154.9, 153.1, 148.9, 147.0, 127.2, 125.2, 123.8, 123.6.

HRMS (ESI): calc. for $C_{12}H_9N_4O_4S^-$ $(M-H)^-$: 305.0350, found: 305.0349.

UV/Vis (LCMS): λ ($\pi \rightarrow \pi^*$) = 328 nm; λ ($n \rightarrow \pi^*$) = 459 nm.

t_R (LCMS; MeCN/H_2O/formic acid = 10/90/0.1 → 90/10/0.1 over 7 min) = 3.871 min.

IR (ATR): wave number/cm^{-1} = 3346, 3261, 3101, 1609, 1540, 1479, 1345, 1323, 1301, 1215, 1156, 1108, 1089, 1004, 896, 858, 842, 754, 730, 718, 683.

m.p. = 215 °C

2.14. (*E*)-4-(Phenyldiazenyl)benzenesulfonamide (1h)

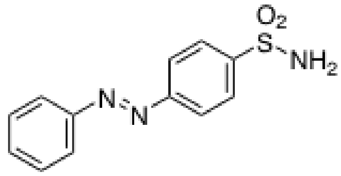

(*E*)-4-(Phenyldiazenyl)benzenesulfonamide was prepared according to procedure A. Flash column chromatography (DCM/MeOH = 100:0 → 100:5) was performed to obtain 291 mg (1.11 mmol, 38%) of the desired product as a red solid.

Amounts: sulfanilamide (1.0 equiv, 0.50 g, 2.90 mmol), nitrosobenzene (1.0 equiv, 311 mg, 2.90 mmol), HOAc (20 mL).

^{1}H NMR (400 MHz, acetone-d_6): δ [ppm] = 8.11–8.05 (m, 2H), 8.05–8.00 (m, 2H), 7.98–7.89 (m, 2H), 7.65–7.51 (m, 2H), 6.71 (br s, 2H).

^{13}C NMR (101 MHz, acetone-d_6): δ [ppm] = 155.0, 153.3, 147.0, 133.0, 130.3, 128.2, 123.9, 123.9.

HRMS (ESI): calc. for $C_{12}H_{10}N_3O_2S^-$ $(M\text{-}H)^-$: 260.0499, found: 260.0498.

UV/Vis (LCMS): λ ($\pi \rightarrow \pi^*$) = 322 nm; λ ($n \rightarrow \pi^*$) = 446 nm.

t_R (LCMS; MeCN/H_2O/formic acid = 10/90/0.1 → 90/10/0.1 over 7 min) = 3.852 min.

IR (ATR): wave number/cm^{-1} = 3353, 3258, 1649, 1583, 1550, 1479, 1439, 1400, 1157, 1143, 1088, 1070, 1020, 1009, 999, 907, 848, 767, 722, 683.

m.p. = 205-207 °C

2.15. (*E*)-Ethyl 4-((4-sulfamoylphenyl)diazenyl)benzoate (1i)

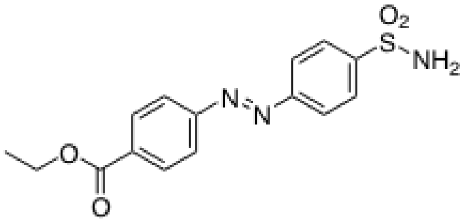

(*E*)-Ethyl 4-((4-sulfamoylphenyl)diazenyl)benzoate was prepared according to procedure A. Flash column chromatography (DCM/MeOH = 100:0 → 100:2) was performed to obtain 46.5 mg (0.14 mmol, quant.) of the desired product as a red solid.

Amounts: sulfanilamide (1.0 equiv, 24.7 mg, 0.14 mmol), ethyl 4-nitrosobenzoate (3.0 equiv, 77.0 mg, 0.43 mmol), HOAc (10 mL).

^{1}H NMR (400 MHz, CD_3OD): δ [ppm] = 8.22 (d, *J* = 8.6 Hz, 2H), 8.14–8.04 (m, 4H), 8.04 (d, *J* = 8.6 Hz, 2H), 4.42 (q, *J* = 7.1 Hz, 2H), 1.43 (t, *J* = 7.1 Hz, 3H).

^{13}C NMR (101 MHz, CD_3OD): δ [ppm] = 167.2, 156.3, 155.5, 147.5, 134.3, 131.7, 128.5, 124.4, 124.0, 62.6, 14.6.

HRMS (ESI): calc. for $C_{15}H_{14}N_3O_4S^-$ $(M-H)^-$: 332.0711, found: 332.0710.

UV/Vis (LCMS): λ ($\pi \rightarrow \pi^*$) = 342 nm; λ ($n \rightarrow \pi^*$) = 455 nm.

$\boldsymbol{t_R}$ (LCMS; MeCN/H_2O/formic acid = 10/90/0.1 → 90/10/0.1 over 7 min) = 4.157 min.

IR (ATR): wave number/cm^{-1} = 3339, 3250, 1708, 1603, 1558, 1408, 1337, 1273, 1216, 1164, 1126, 1105, 1093, 1025, 1008, 901, 864, 847, 830, 772, 726, 717, 691, 654.

m.p. = 190 °C

2.16. Sodium (phenylamino)methanesulfonate hydrate (2)

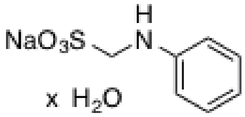

Sodium (phenylamino)methanesulfonate hydrate was synthesized according to a literature procedure [2]. Briefly, a round bottomed flask was charged with $NaHSO_3$ (1.0 equiv, 5.72 g, 55.0 mmol) and formalin (37%, 1.0 equiv, 4.46 g, 4.1 mL, 55.0 mmol) in 100 mL H_2O and warmed to 40 °C before aniline (1.0 equiv, 5.11 g, 55.0 mmol) was added dropwise. The solution was stirred at 50 °C for 3 h before it was concentrated to ~1/10 of its initial volume. The resulting crystalline solid was filtered off and washed with 200 mL of a MeOH/EtOH mixture (10:90). The crystals were dried under HV and analytically pure for X-ray crystallography.

^{1}H NMR (400 MHz, DMSO-d_6): δ [ppm] = 7.01 (t, J = 7.7 Hz, 2H), 6.69 (d, J = 8.0 Hz, 2H), 6.58–6.43 (m, 1H), 5.94 (t, J = 6.8 Hz, 1H), 3.88 (d, J = 6.7 Hz, 2H).

^{13}C NMR (101 MHz, DMSO-d_6): δ [ppm] = 148.1, 128.5, 115.7, 112.5, 60.6.

HRMS (ESI): calc. for $C_7H_8NO_3S^-$ (M-Na)$^-$: 186.0230, found: 186.0230.

UV/Vis (LCMS): λ_{max} = 242 nm, 290 nm.

t_R (LCMS; MeCN/H_2O/formic acid = 10/90/0.1 → 100/0/0.1 over 10 min) = 2.797 min.

IR (ATR): wave number/cm^{-1} = 3463, 3319, 1645, 1601, 1517, 1499, 1441, 1420, 1320, 1254, 1225, 1207, 1161, 1036, 880, 752, 745, 688.

m.p. = decomposition >243 °C

3. *Spectra*

3.1. (*E*)-4-(4-Hydroxyphenyldiazenyl)benzenesulfonamide (1a)

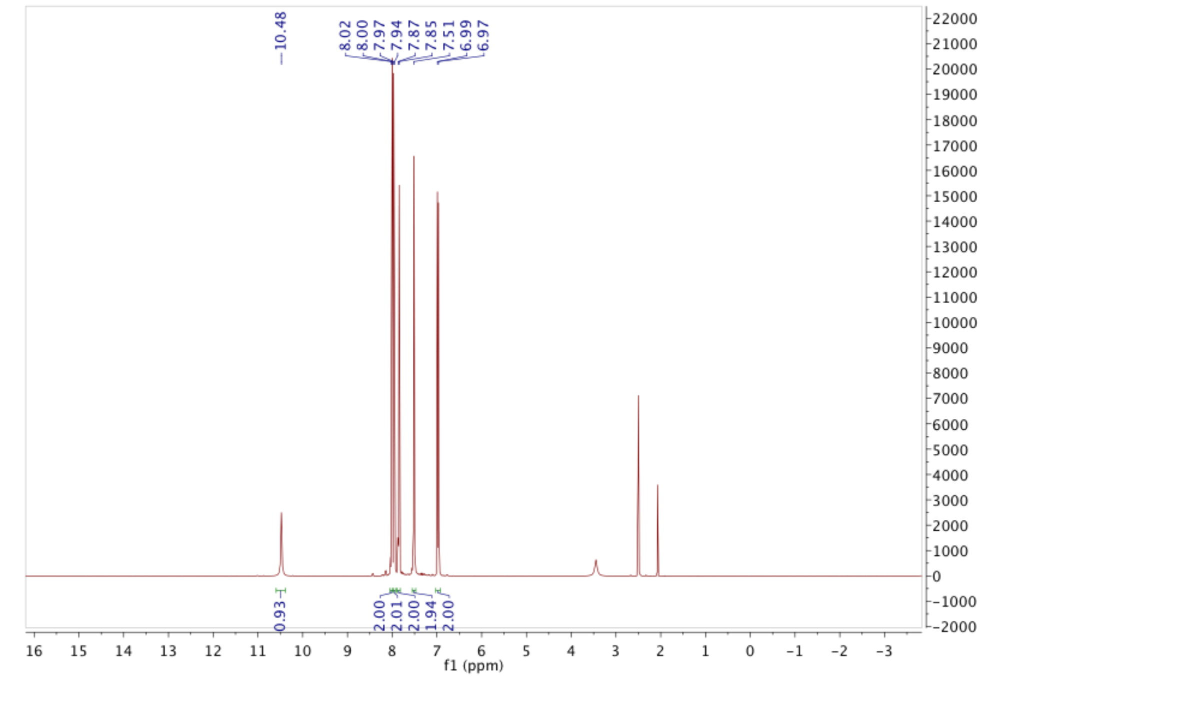

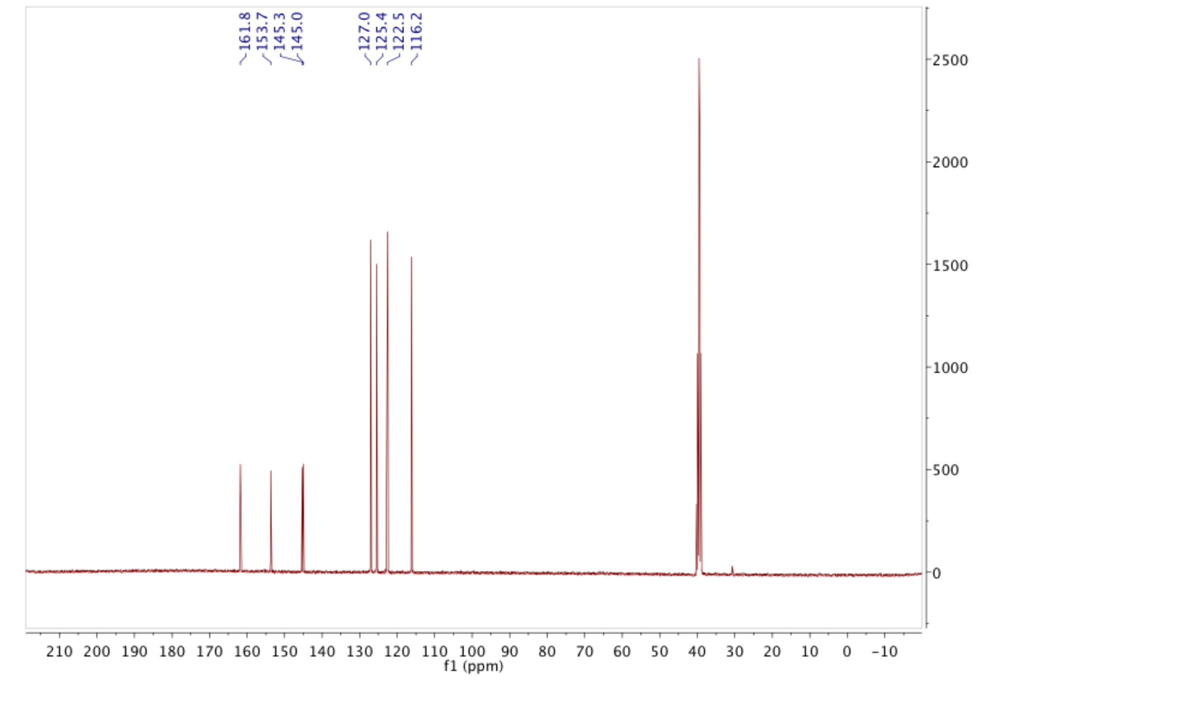

3.2. (*E*)-4-((4-(Diethylamino)phenyl)diazenyl)benzenesulfonamide (1b)

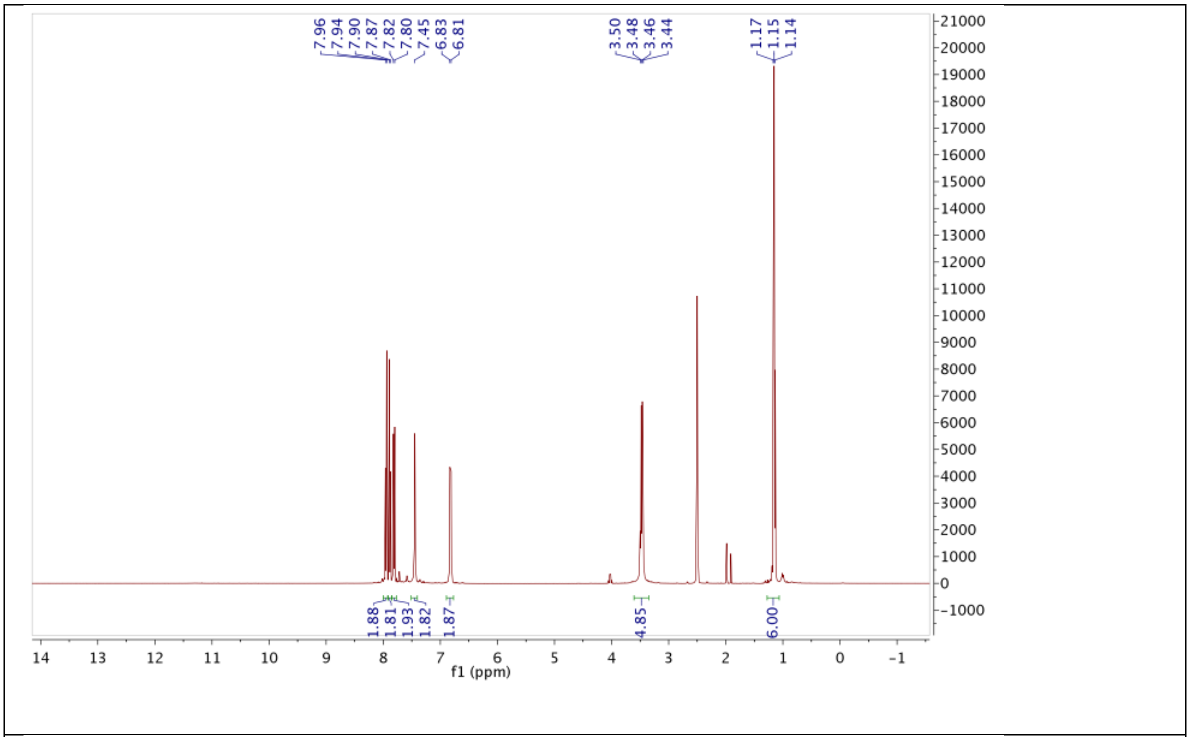

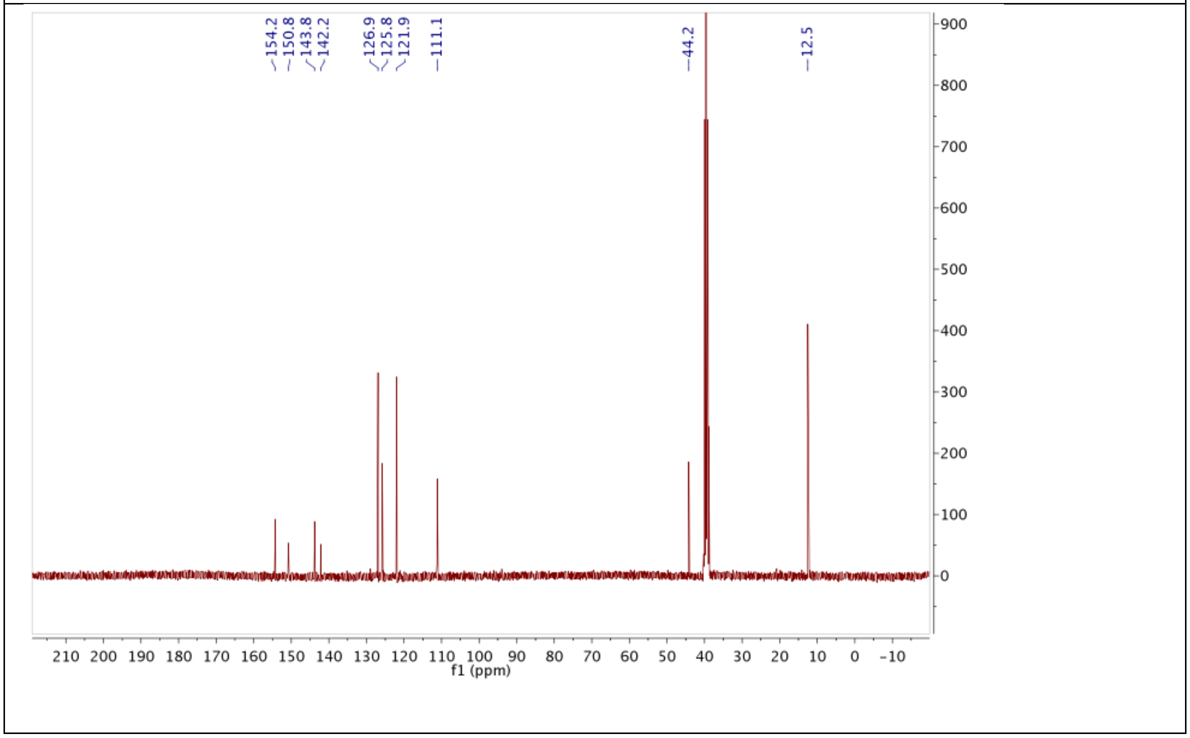

3.3. (*E*)-4-((4-Morpholinophenyl)diazenyl)benzenesulfonamide (1c)

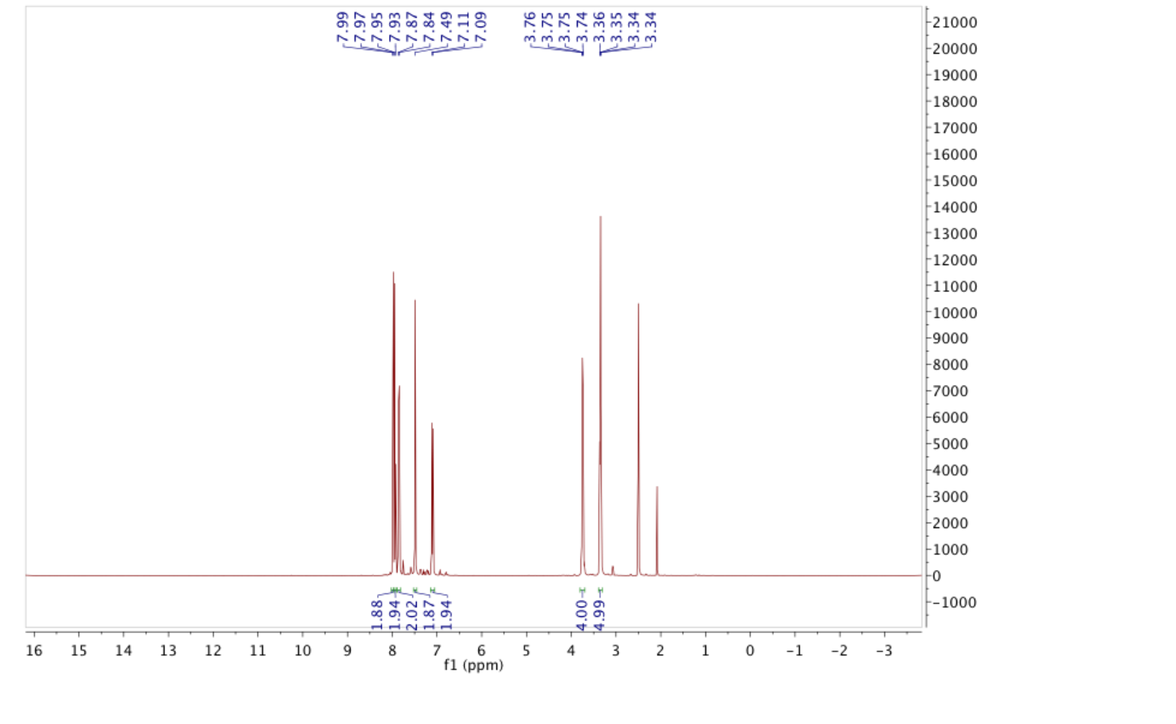

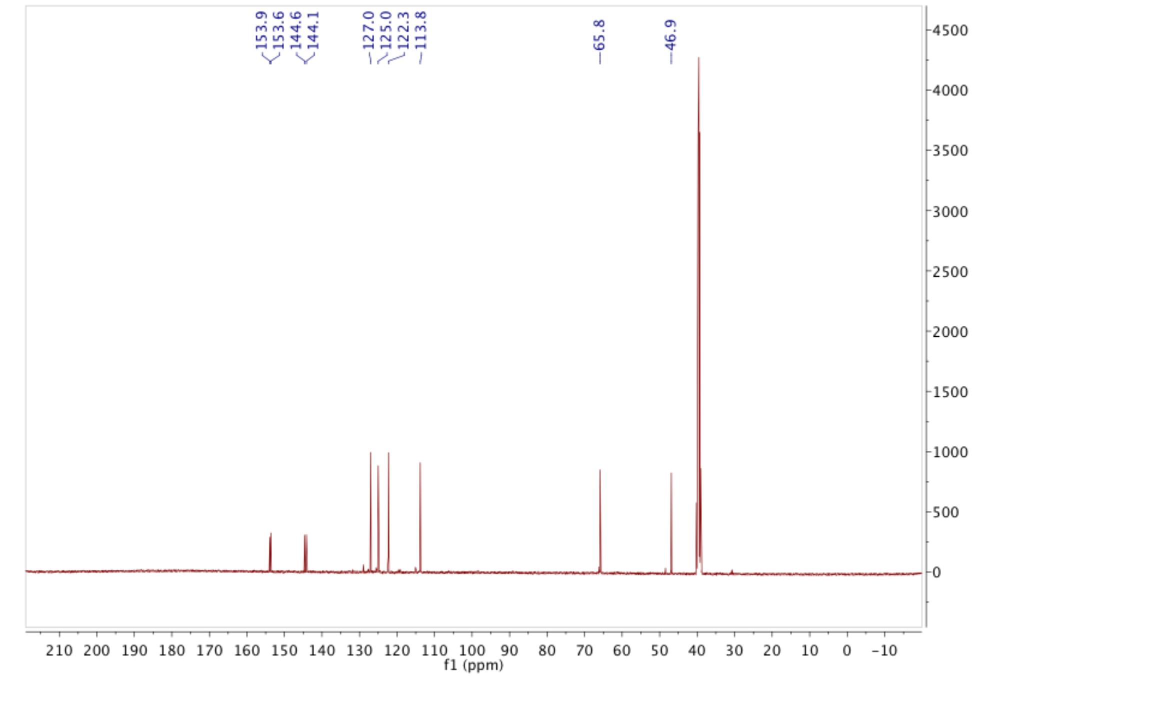

3.4. (*E*)-4-(4-Aminophenyldiazenyl)benzenesulfonamide (1d)

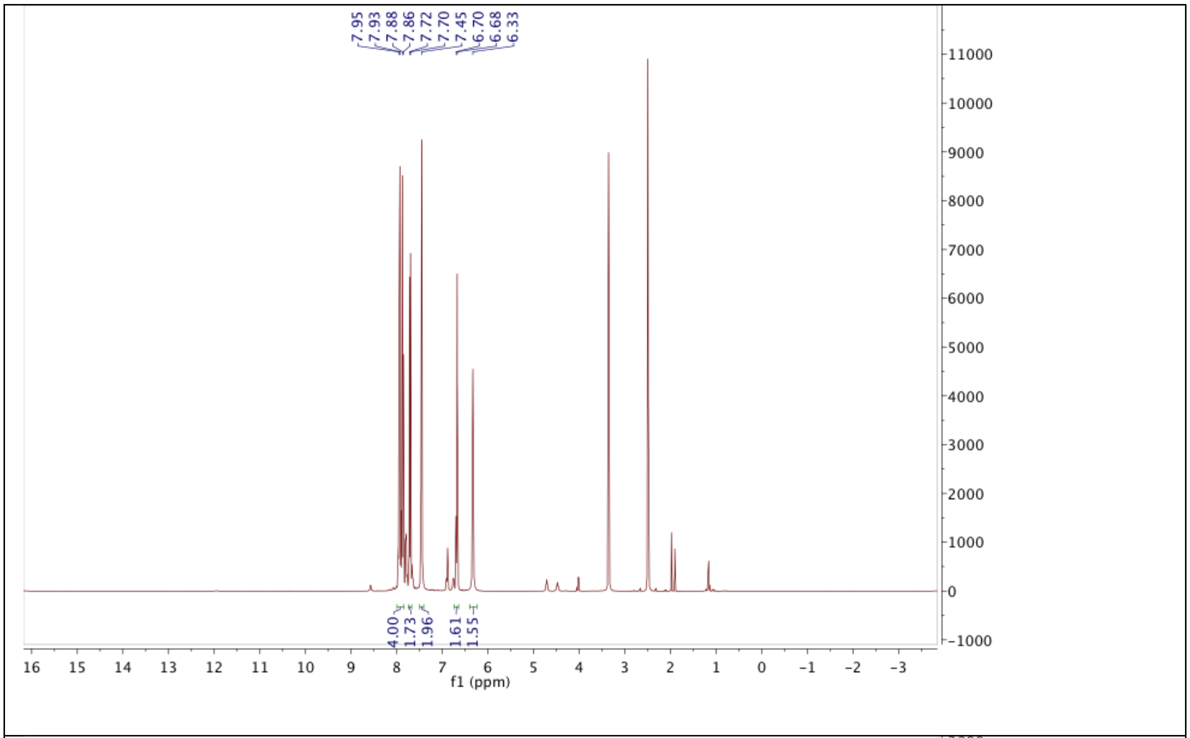

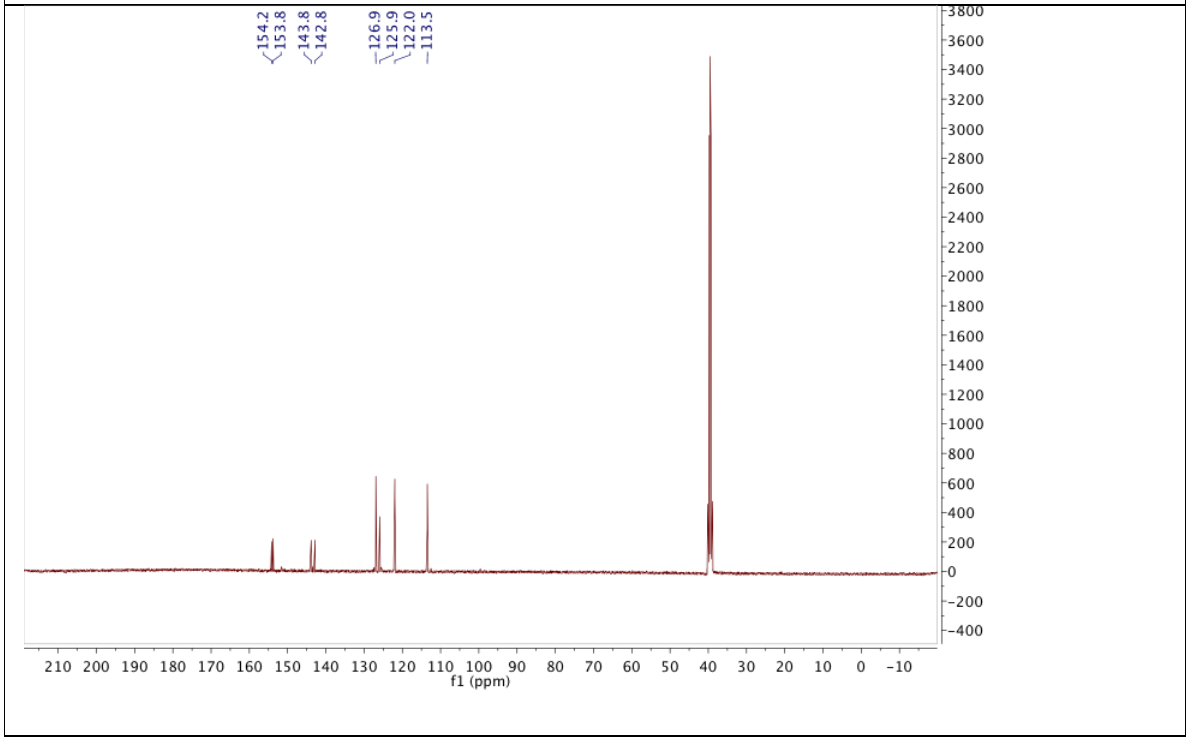

3.5. (*E*)-4-(4-Azidophenyldiazenyl)benzenesulfonamide (1e)

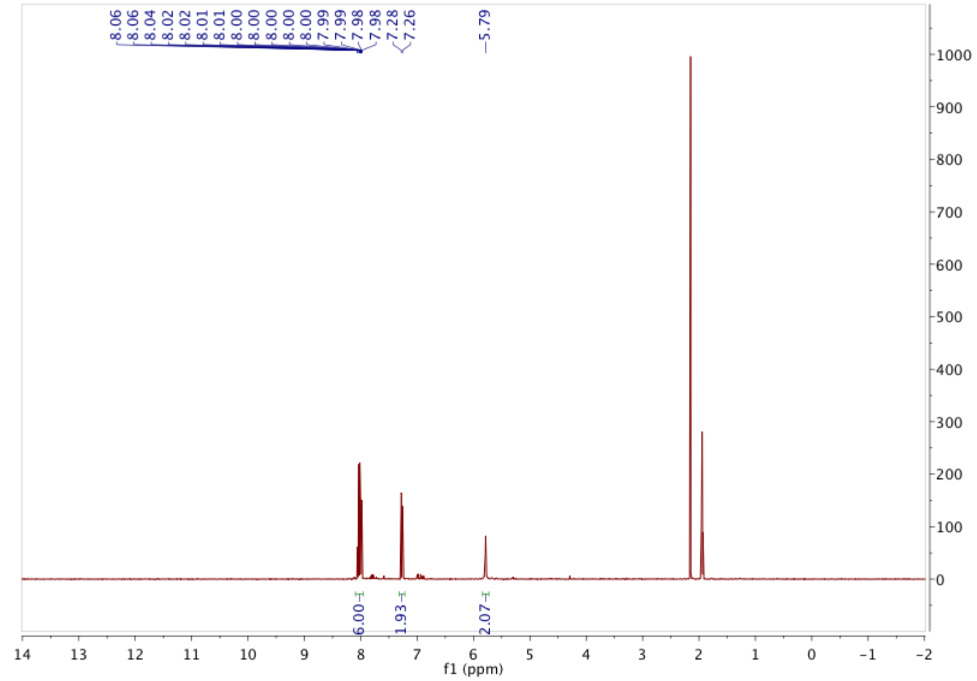

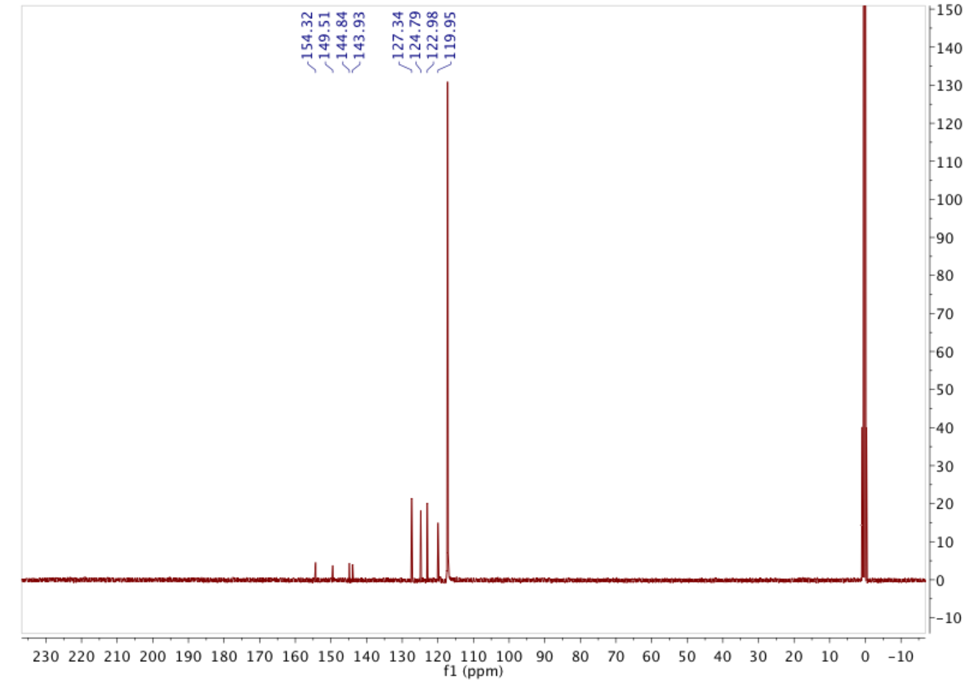

3.6. (*E*)-4-(*p*-Tolyldiazenyl)benzenesulfonamide (1f)

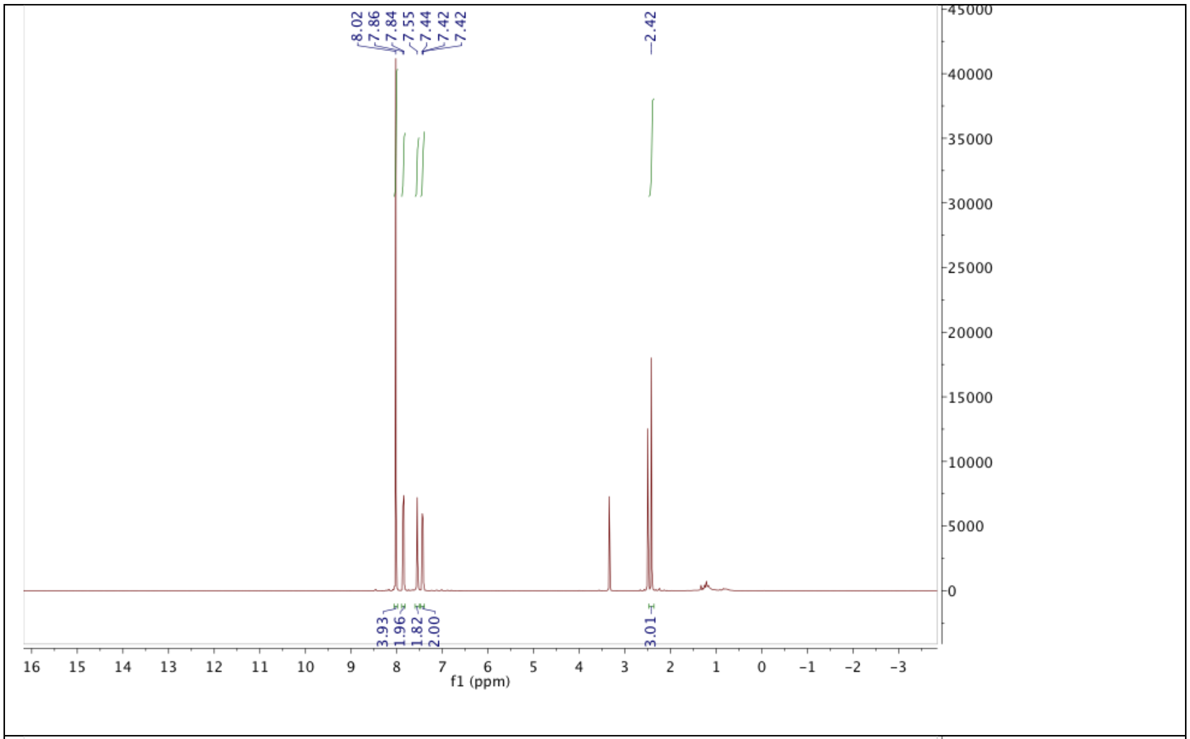

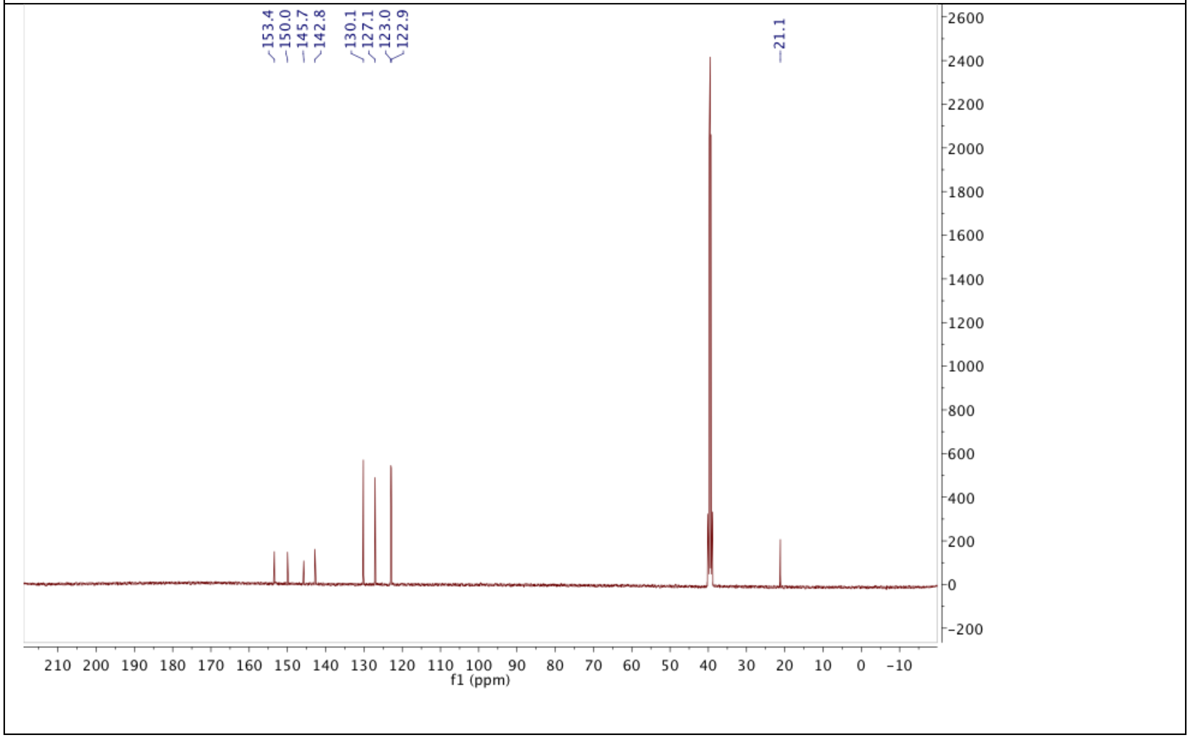

3.7. (*E*)-4-((4-Nitrophenyl)diazenyl)benzenesulfonamide (1g)

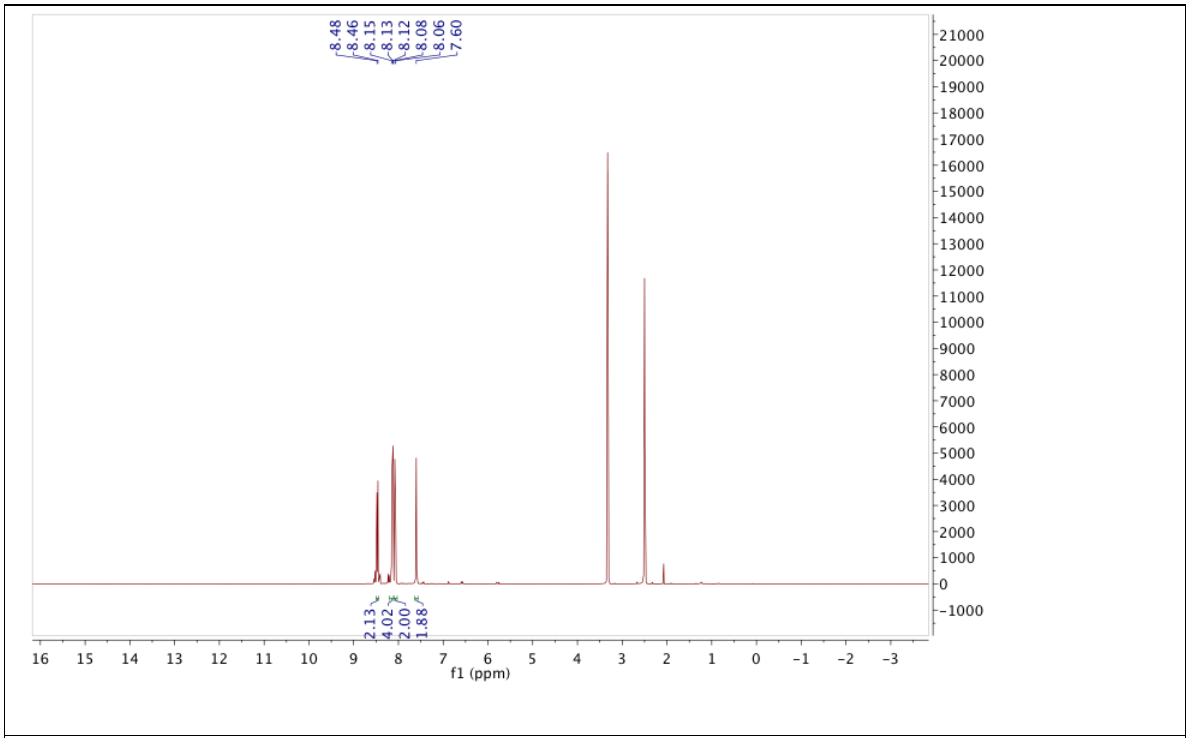

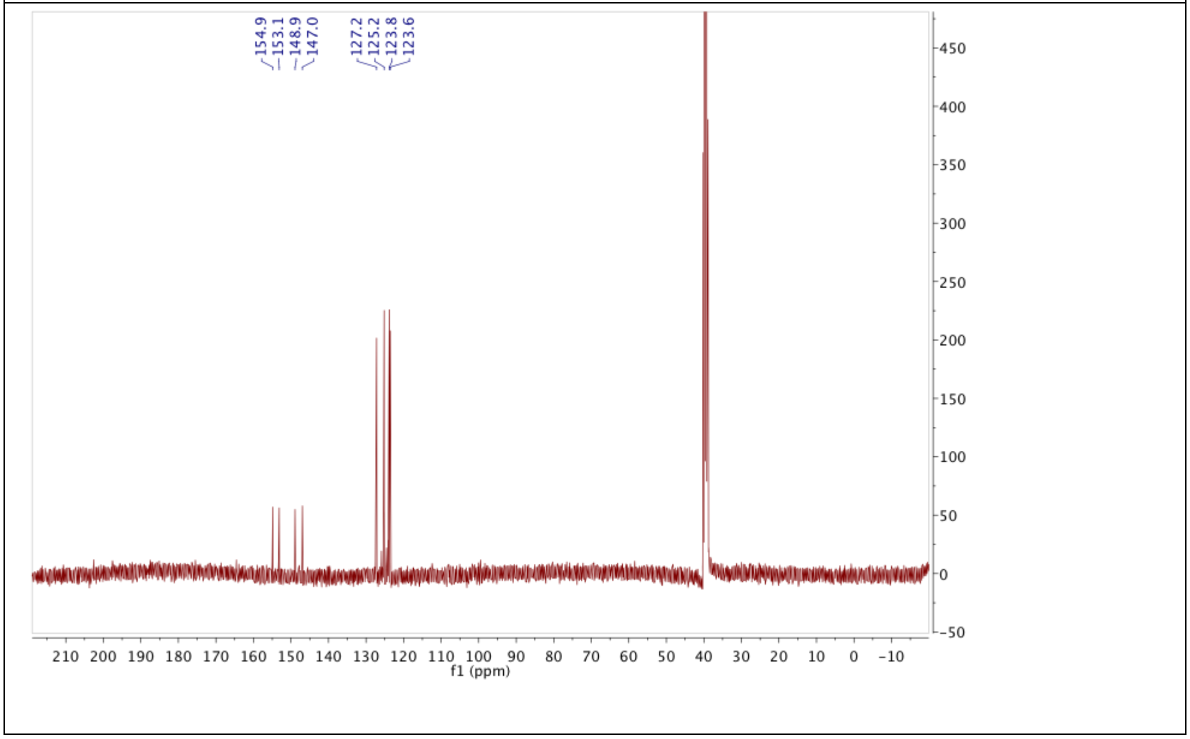

3.8. (*E*)-4-(Phenyldiazenyl)benzenesulfonamide (1h)

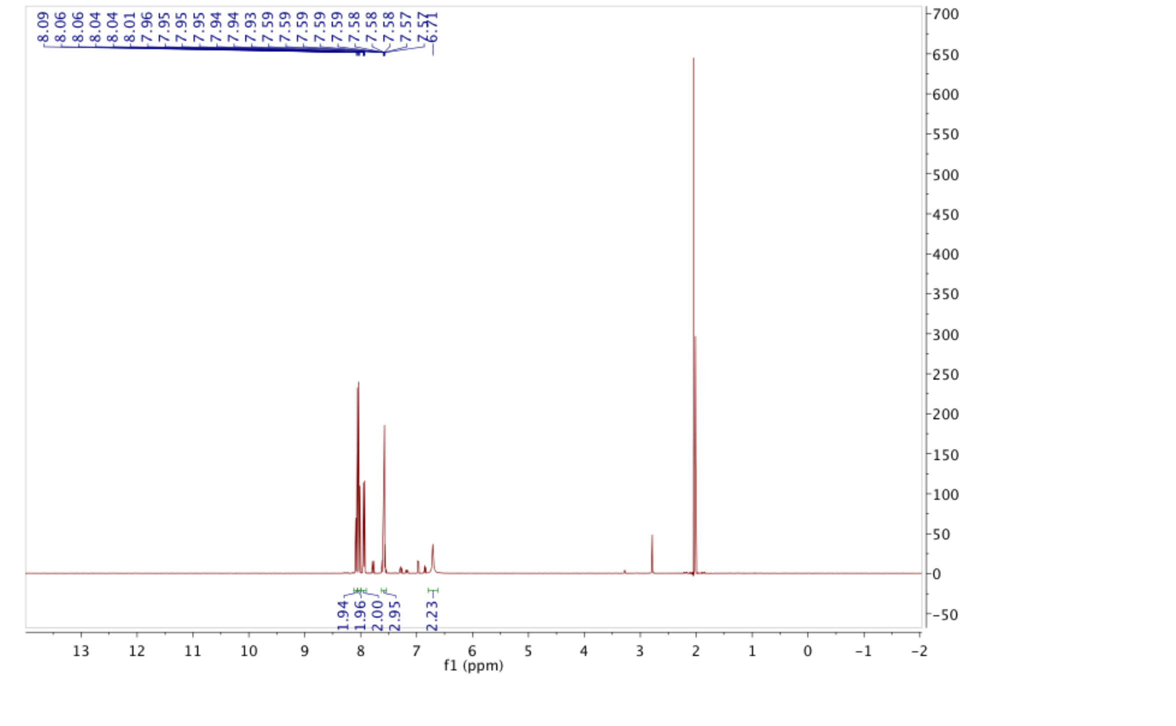

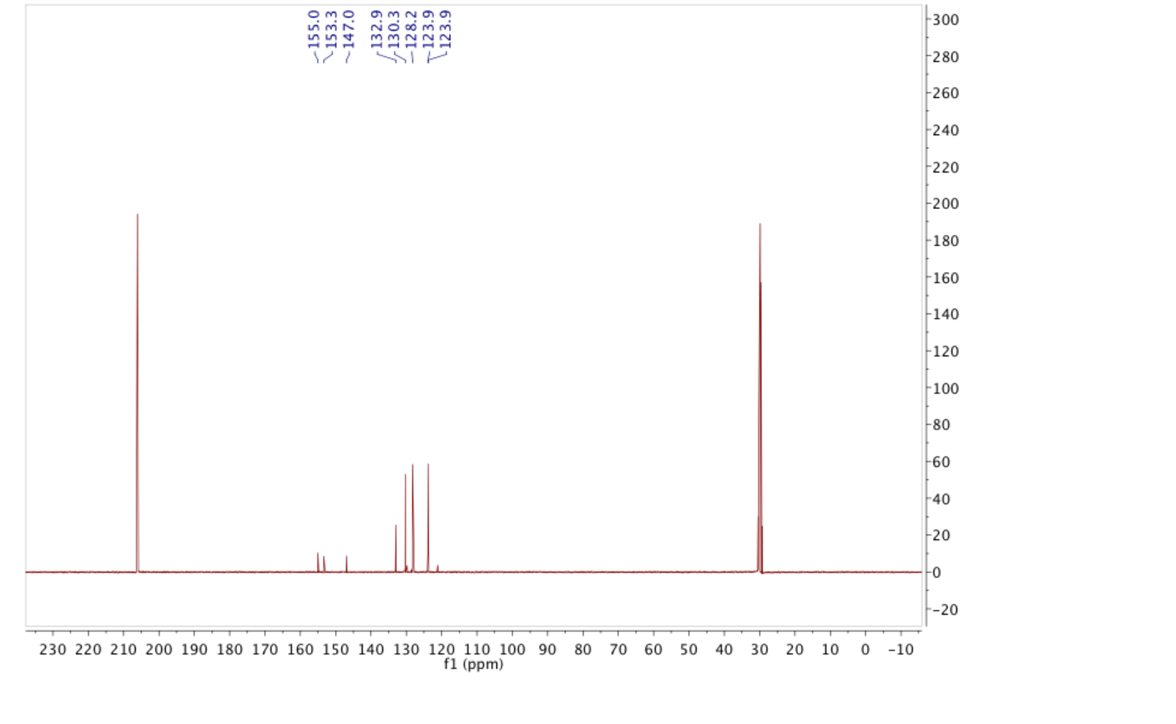

3.9. (*E*)-Ethyl 4-((4-sulfamoylphenyl)diazenyl)benzoate (1i)

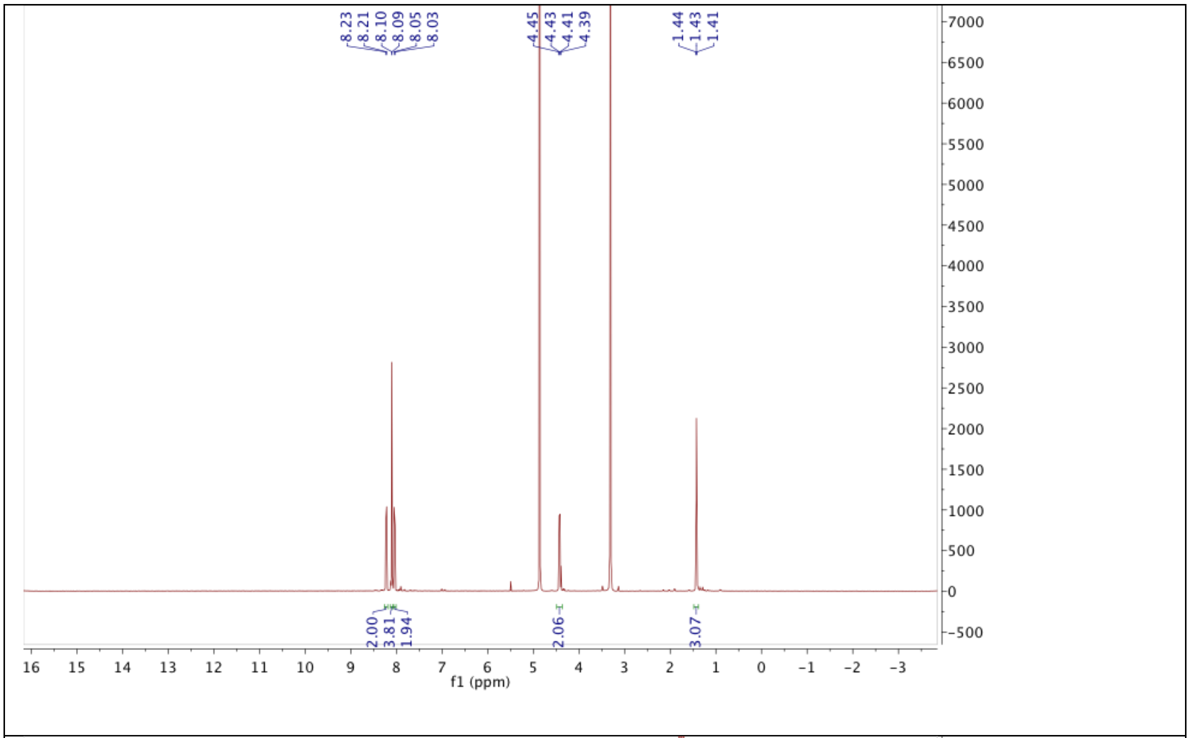

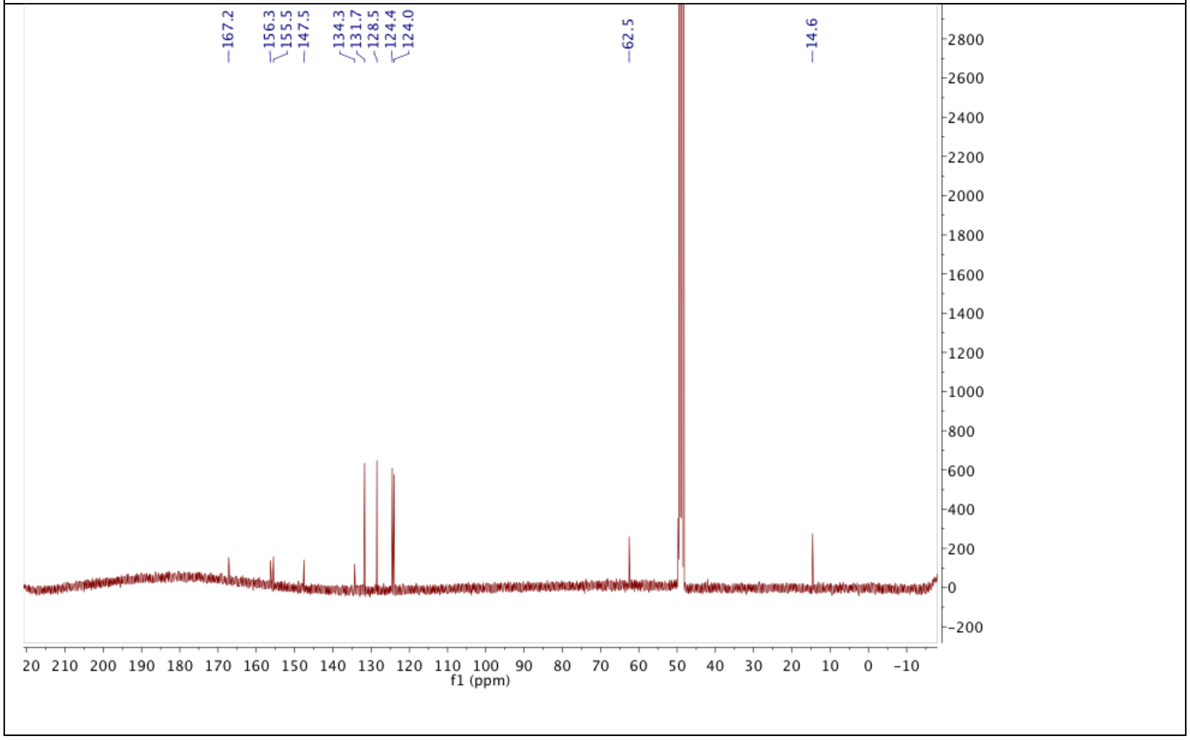

3.10. Sodium (phenylamino)methanesulfonate hydrate (2)

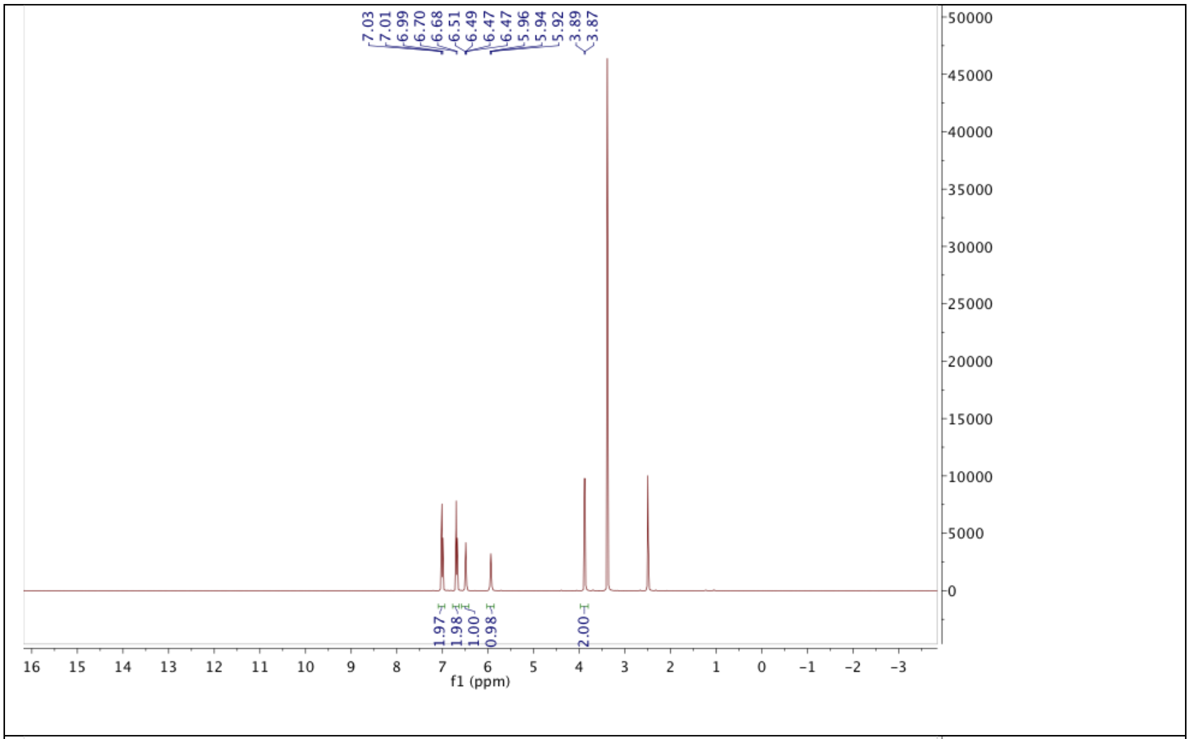

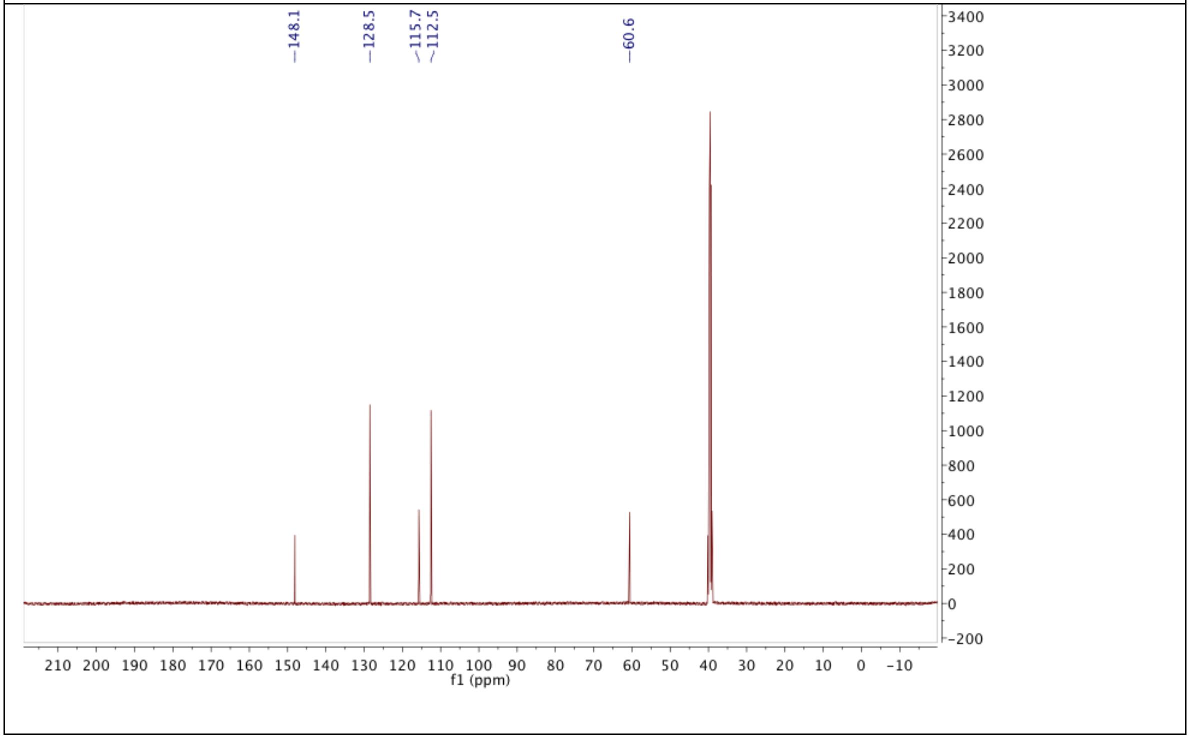

4. X-Ray crystallographic data

Crystals suitable for X-Ray diffractometry for **1a** were obtained from a DMSO/water solution. Crystals suitable for X-Ray diffractometry for **1b**, **1c**, **1e**, **1f** and **1g** were obtained by allowing a concentrated solution of the azobenzene in DMSO to stand open to the atmosphere for 2-10 days. Crystals suitable for X-ray diffractometry for **1d** were obtained from a 1 M HCl/EtOH solution. Crystals suitable for X-ray diffractometry for **1h** were obtained from an EtOAc solution. Crystals suitable for X-ray diffractometry for **1i** were obtained from a hot solution of EtOH.

All structures have been solved by direct methods with SIR97 with the exception of **1a** (SHELXS). The refinements were performed with SHELXL. C-bound hydrogen atoms have been added in ideally calculated positions riding on their parent atoms in all structures while all parameters of N- and O-bound hydrogen atoms have been refined freely with the exception of the N-bound hydrogen atoms in **1h**. They have been refined as the C-bound hydrogen atoms. Water bound hydrogen atoms in **1f** have not been considered in the refinement.

The dataset from the complex of hCAII bound to **1d** was collected using synchrotron radiation (λ = 1.0 Å) at the X06SA-beamline (Swiss Light Source, Villingen, Switzerland). X-ray intensities and data reduction were evaluated using the XDS program package [3] (Supplementary Table 11). Notably, crystals diffracted better than 1.0 Å resolution, however the collection protocol with the installed Pilatus detector only allowed a maximum resolution of 1.15 with a high resolution bin of being > 90% complete. Conventional crystallographic rigid body, positional, and temperature factor refinements were carried out with REFMAC5 [4] using coordinates of the human carbonic anhydrase structure as starting model (PDB ID code 2VVA[5]). For model building, the programs SYBYL and MAIN [6] were used. The final coordinates yielded excellent R factors, as well as RMSD bond and angle values. Coordinates were confirmed to fulfill the Ramachandran plot and have been deposited in the RCSB (5BYI). Molecular illustrations were prepared in PyMOL (DeLano Scientific, Palo Alto, CA, USA).

4.1. (*E*)-4-((4-(Hydroxyphenyl)diazenyl)benzenesulfonamide (1a)

Supplementary Table 1:Crystallographic data of **1a**.

	**1a**
net formula	$C_{12}H_{11}N_3O_3S$
M_r/g mol^{-1}	277.300
crystal size/mm	0.090 × 0.060 × 0.040
T/K	173(2)
radiation	'Mo Kα
diffractometer	'Bruker D8Venture'
crystal system	triclinic
space group	*P*1
a/Å	5.9930(2)
b/Å	7.7671(3)
c/Å	26.2903(11)
α/°	81.8058(13)
β/°	83.7090(13)
γ/°	89.4342(12)
V/Å^3	1203.96(8)
Z	4
calc. density/g cm^{-3}	1.52987(10)
μ/mm^{-1}	0.277
absorption correction	multi-scan
transmission factor range	0.9074–0.9585
refls. measured	21464
R_{int}	0.0273
mean σ(*I*)/*I*	0.0377
θ range	2.36–26.40

observed refls.	7359
x, *y* (weighting scheme)	0.0457, 0.2288
hydrogen refinement	mixed
Flack parameter	0.02(4)
refls in refinement	8998
parameters	733
restraints	15
$R(F_{obs})$	0.0374
$R_w(F^2)$	0.0929
S	1.045
shift/error$_{max}$	0.001
max electron density/e Å^{-3}	0.250
min electron density/e Å^{-3}	−0.258
CDCC	1041473

C-H: constr, N-H und O-H: refall

4.2. (*E*)-4-((4-(Diethylamino)phenyl)diazenyl)benzenesulfonamide (1b)

Supplementary Table 2: Crystallographic data of **1b**.

	**1b**
net formula	$C_{18}H_{26}N_4O_3S_2$
M_r/g mol^{-1}	410.556
crystal size/mm	0.060 × 0.050 × 0.040
T/K	100(2)
radiation	'Mo Kα
diffractometer	'Bruker D8Venture'
crystal system	monoclinic
space group	$P2_1$
a/Å	7.5368(3)
b/Å	8.1064(3)
c/Å	16.7424(7)
α/°	90
β/°	94.6119(14)
γ/°	90
V/Å^3	1019.59(7)
Z	2
calc. density/g cm^{-3}	1.33731(9)
μ/mm^{-1}	0.287
absorption correction	multi-scan
transmission factor range	0.8939–0.9585
refls. measured	12699
R_{int}	0.0255
mean σ(*I*)/*I*	0.0283
θ range	3.06–26.49
observed refls.	3925
x, *y* (weighting scheme)	0.0312, 0.2439
hydrogen refinement	mixed

Flack parameter	−0.01(4)
refls in refinement	4138
parameters	256
restraints	1
$R(F_{obs})$	0.0253
$R_w(F^2)$	0.0623
S	1.051
shift/error$_{max}$	0.001
max electron density/e Å^{-3}	0.310
min electron density/e Å^{-3}	−0.281
CDCC	1041468

4.3. (*E*)-4-((4-Morpholinophenyl)diazenyl)benzenesulfonamide (1c)

Supplementary Table 3: Crystallographic data of **1c**.

	**1c**
net formula	$C_{16}H_{18}N_4O_3S$
M_r/g mol^{-1}	346.405
crystal size/mm	0.130 × 0.120 × 0.040
T/K	100(2)
radiation	'Mo Kα
diffractometer	'Bruker D8Venture'
crystal system	orthorhombic
space group	$Pna2_1$
a/Å	22.6988(7)
b/Å	8.4921(3)
c/Å	25.0001(8)
α/°	90
β/°	90
γ/°	90
V/Å^3	4819.0(3)
Z	12
calc. density/g cm^{-3}	1.43240(9)
μ/mm^{-1}	0.225
absorption correction	multi-scan
transmission factor range	0.9040–0.9585
refls. measured	81173
R_{int}	0.0342
mean σ(I)/I	0.0198
θ range	3.00–26.41
observed refls.	9161
x, y (weighting scheme)	0.0425, 1.4290
hydrogen refinement	mixed

Flack parameter	0.09(4)
refls in refinement	9848
parameters	682
restraints	1
$R(F_{obs})$	0.0305
$R_w(F^2)$	0.0765
S	1.033
shift/error$_{max}$	0.001
max electron density/e Å^{-3}	0.328
min electron density/e Å^{-3}	−0.264
CDCC	1041466

4.4. (*E*)-4-(4-Aminophenyldiazenyl)benzenesulfonamide (1d)

Supplementary Table 4: Crystallographic data of **1d**.

	**1d**
net formula	$C_{12}H_{14}N_4O_3S$
M_r/g mol^{-1}	294.331
crystal size/mm	0.120 × 0.080 × 0.040
T/K	173(2)
radiation	'Mo Kα
diffractometer	'Bruker D8Venture'
crystal system	orthorhombic
space group	$P2_12_12$
a/Å	7.2743(3)
b/Å	30.3691(13)
c/Å	6.0082(2)
α/°	90
β/°	90
γ/°	90
V/Å^3	1327.30(9)
Z	4
calc. density/g cm^{-3}	1.47293(10)
μ/mm^{-1}	0.258
absorption correction	multi-scan
transmission factor range	0.9053–0.9585
refls. measured	14363
R_{int}	0.0266
mean σ(I)/I	0.0211
θ range	3.10–26.44
observed refls.	2517
x, y (weighting scheme)	0.0467, 0.3027
hydrogen refinement	mixed

Flack parameter	−0.01(8)
refls in refinement	2716
parameters	205
restraints	0
$R(F_{obs})$	0.0334
$R_w(F^2)$	0.0841
S	1.102
shift/error$_{max}$	0.001
max electron density/e Å^{-3}	0.302
min electron density/e Å^{-3}	−0.193
CDCC	1041472

4.5. (*E*)-4-(4-Azidophenyldiazenyl)benzenesulfonamide (1e)

Supplementary Table 5: Crystallographic data of **1e**.

	**1e**
net formula	$C_{14}H_{16}N_6O_3S_2$
M_r/g mol^{-1}	380.448
crystal size/mm	0.130 × 0.110 × 0.090
T/K	173(2)
radiation	'Mo Kα
diffractometer	'Bruker D8Venture'
crystal system	monoclinic
space group	$P2_1/c$
a/Å	9.5471(3)
b/Å	6.9479(3)
c/Å	26.7173(10)
α/°	90
β/°	97.7199(11)
γ/°	90
V/Å^3	1756.16(11)
Z	4
calc. density/g cm^{-3}	1.43895(9)
μ/mm^{-1}	0.330
absorption correction	multi-scan
transmission factor range	0.8804–0.9590
refls. measured	21511
R_{int}	0.0246
mean σ(I)/I	0.0201
θ range	3.03–27.55
observed refls.	3437
x, y (weighting scheme)	0.0592, 1.1238
hydrogen refinement	mixed

refls in refinement	4019
parameters	236
restraints	0
$R(F_{obs})$	0.0401
$R_w(F^2)$	0.1118
S	1.017
shift/error$_{max}$	0.001
max electron density/e Å^{-3}	0.967
min electron density/e Å^{-3}	−0.373
CDCC	1041469

4.6. (*E*)-4-(*p*-Tolyldiazenyl)benzenesulfonamide (1f)

Supplementary Table 6: Crystallographic data of **1f**.

	**1f**
net formula	$C_{14.70}H_{18.40}N_3O_3S_{1.85}$
M_r/g mol^{-1}	344.444
crystal size/mm	0.090 × 0.060 × 0.010
T/K	173(2)
radiation	'Mo Kα
diffractometer	'Bruker D8Venture'
crystal system	monoclinic
space group	$P2_1/c$
a/Å	9.5107(4)
b/Å	6.6459(3)
c/Å	27.6033(11)
α/°	90
β/°	95.6025(11)
γ/°	90
V/Å^3	1736.39(13)
Z	4
calc. density/g cm^{-3}	1.31761(10)
μ/mm^{-1}	0.304
absorption correction	multi-scan
transmission factor range	0.9048–0.9585
refls. measured	28311
R_{int}	0.0333
mean σ(*I*)/*I*	0.0215
θ range	2.97–26.40
observed refls.	2811
x, *y* (weighting scheme)	0.0658, 1.0650
hydrogen refinement	mixed

refls in refinement	3563
parameters	220
restraints	0
$R(F_{obs})$	0.0429
$R_w(F^2)$	0.1345
S	1.107
shift/error$_{max}$	0.001
max electron density/e Å^{-3}	0.600
min electron density/e Å^{-3}	−0.400
CDCC	1041467

4.7. (*E*)-4-((4-Nitrophenyl)diazenyl)benzenesulfonamide (1g)

Supplementary Table 7: Crystallographic data of **1g**.

	**1g**
net formula	$C_{14}H_{16}N_4O_5S_2$
M_r/g mol^{-1}	384.433
crystal size/mm	0.120 × 0.080 × 0.050
T/K	173(2)
radiation	'Mo Kα
diffractometer	'Bruker D8Venture'
crystal system	monoclinic
space group	$P2_1/c$
a/Å	9.7420(5)
b/Å	6.9706(3)
c/Å	25.6450(13)
α/°	90
β/°	95.7761(14)
γ/°	90
V/Å^3	1732.65(15)
Z	4
calc. density/g cm^{-3}	1.47375(13)
μ/mm^{-1}	0.341
absorption correction	multi-scan
transmission factor range	0.9006–0.9585
refls. measured	17953
R_{int}	0.0242
mean σ(*I*)/*I*	0.0216
θ range	2.76–26.40
observed refls.	2924
x, *y* (weighting scheme)	0.0667, 1.5179
hydrogen refinement	mixed

refls in refinement	3554
parameters	236
restraints	0
$R(F_{obs})$	0.0501
$R_w(F^2)$	0.1396
S	1.054
shift/error$_{max}$	0.001
max electron density/e Å^{-3}	0.545
min electron density/e Å^{-3}	−0.306
CDCC	1041470

4.8. (*E*)-4-((Phenyl)diazenyl)benzenesulfonamide (1h)

Supplementary Table 8: Crystallographic data of **1h**.

	**1h**
net formula	$C_{12}H_{11}N_3O_2S$
M_r/g mol^{-1}	261.30
crystal size/mm	0.120 × 0.100 × 0.010
T/K	100(2)
radiation	MoKα
diffractometer	'Bruker D8Venture'
crystal system	monoclinic
space group	'P c'
a/Å	13.5558(11)
b/Å	5.3493(5)
c/Å	8.1371(7)
α/°	90
β/°	90.687(3)
γ/°	90
V/Å^3	590.01(9)
Z	2
calc. density/g cm^{-3}	1.471
μ/mm^{-1}	0.271
absorption correction	multi-scan
transmission factor range	0.8853–0.9585
refls. measured	10023
R_{int}	0.0447
mean σ(*I*)/*I*	0.0422
θ range	3.005–26.40

observed refls.	1995
x, y (weighting scheme)	0.0496, 0.2150
hydrogen refinement	constr
Flack parameter	−0.03(13)
refls in refinement	2289
parameters	164
restraints	2
$R(F_{obs})$	0.0409
$R_w(F^2)$	0.0949
S	1.033
shift/error$_{max}$	0.001
max electron density/e Å^{-3}	0.271
min electron density/e Å^{-3}	−0.293
CDCC	1041813

4.9. (*E*)-Ethyl 4-((4-sulfamoylphenyl)diazenyl)benzoate (1i)

Supplementary Table 9: Crystallographic data of **1i**.

	**1i**
net formula	$C_{15}H_{15}N_3O_4S$
M_r/g mol^{-1}	333.363
crystal size/mm	0.150 × 0.030 × 0.020
T/K	173(2)
radiation	'Mo Kα
diffractometer	'Bruker D8Venture'
crystal system	monoclinic
space group	$P2_1/n$
a/Å	16.4622(12)
b/Å	4.9986(3)
c/Å	18.5166(12)
α/°	90
β/°	90.449(2)
γ/°	90
V/Å^3	1523.65(17)
Z	4
calc. density/g cm^{-3}	1.45328(16)
μ/mm^{-1}	0.237
absorption correction	multi-scan
transmission factor range	0.8535–0.9585
refls. measured	17872
R_{int}	0.0618
mean σ(I)/I	0.0485
θ range	3.30–26.40
observed refls.	2249
x, y (weighting scheme)	0.0377, 0.9958
hydrogen refinement	mixed

refls in refinement	3115
parameters	217
restraints	0
$R(F_{obs})$	0.0421
$R_w(F^2)$	0.0988
S	1.048
shift/error$_{max}$	0.001
max electron density/e Å^{-3}	0.530
min electron density/e Å^{-3}	−0.382
CCDC	1041471

4.10. Sodium (phenylamino)methanesulfonate hydrate (2)

Supplementary Table 10: Crystallographic data of **2**.

	**2**
net formula	$C_7H_{10}NNaO_4S$
M_r/g mol^{-1}	227.214
crystal size/mm	0.266 × 0.168 × 0.063
T/K	173(2)
radiation	MoKα
diffractometer	'Oxford XCalibur'
crystal system	orthorhombic
space group	'P 21 21 21'
a/Å	5.3116(3)
b/Å	5.8953(2)
c/Å	29.8056(12)
α/°	90
β/°	90
γ/°	90
V/Å^3	933.33(7)
Z	4
calc. density/g cm^{-3}	1.61702(12)
μ/mm^{-1}	0.379
absorption correction	'multi-scan'
transmission factor range	0.98233–1.00000
refls. measured	5022
R_{int}	0.0391
mean σ(I)/I	0.0514
θ range	4.351–26.364

observed refls.	1722
x, *y* (weighting scheme)	0.0306, 0.00
hydrogen refinement	mixed
Flack parameter	−0.01(6)
refls in refinement	1899
parameters	139
restraints	3
$R(F_{obs})$	0.0337
$R_w(F^2)$	0.0729
S	1.042
shift/error$_{max}$	0.001
max electron density/e Å^{-3}	0.249
min electron density/e Å^{-3}	−0.294
CCDC	1041814

C-H: constr, N- und O-H: refall.

4.11. wt hCAII bound to 1d

Supplementary Table 11: Crystallographic data collection and refinement statistics of the hCAII in complex with **1d**

Crystallographic data	hCAII:**1d***
**Crystal parameters**	
Space group	$P2_1$
Cell constants (Å/°)	a = 42.1
(1 hCAII per AU)	b = 41.2
	c = 71.9
	β = 104.2
**Data collection**	
Beamline	X06SA, SLS
Wavelength, Å	1.0
Resolution range, Å†	30-1.25 (1.25-1.15)
No. observations	345817
No. unique reflections‡	82060
Completeness, %†	96.5 (92.5)
R_{merge}†,§	0.056 (0.078)
I/σ (I)†	17.3 (10.6)

Refinement

Resolution range, (Å)	10-1.15
No. reflections working set	77957
No. reflections test set	4103
No. nonhydrogen	2437
No. of ligand atoms	19
Solvent molecules (H_2O, Zn^{2+}, K^+, Hg^{2+}, …)	358
R_{work}/R_{free} (%)¶	17.1/18.7
Rmsd bond (Å)/(°)**	0.008/1.296
Ramachandran plot, %***	95.7/4.3/0
PDB accession code	5BYI

*Dataset has been collected on a single crystal
†Values in parentheses of resolution range, completeness, Rmerge, and I/σ (I) correspond to the last resolution shell.
‡Friedel pairs were treated as identical reflections
§Rmerge(I) = $\Sigma_{hkl}\Sigma_j$ | $I(hkl)_j$ - <I(hkl)> | / Σ_{hkl} Σ_j $I(hkl)_j$, where $I(hkl)_j$ is the jth measurement of the intensity of reflection hkl and <I(hkl)> is the average intensity
¶$R = \Sigma_{hkl}||F_{obs}| - |F_{calc}||/\Sigma_{hkl}|F_{obs}|$, where R_{free} is calculated without a sigma cut off for a randomly chosen 5% of reflections, which were not used for structure refinement, and R_{work} is calculated for the remaining reflections
**Deviations from ideal bond lengths/angles
***Number of residues in favored region/allowed region/outlier region

5. hCAII purification, crystallization and assay

5.1. Purification and crystallization

Wild-type human Carbonic Anhydrase II (hCAII) was expressed and purified as previously reported [2,7]. A 5 L culture yielded 100 mg of >95% pure protein, confirmed by SDS-PAGE. Prior to crystallization, hCAII was further purified by gel filtration using an AEKTA purifier with gel filtration buffer as an eluent. Pure hCAII was dissolved in H_2O (10 mg/mL) and centrifuged for 25 min at 13,500 rpm at 4 °C. The supernatant was separated into 49 µL aliquots and 1 µL of ligand (69 mM in DMSO) was added. After sufficient incubation at rt, the resulting suspension was centrifuged for 25 min at 13,500 rpm at 4 °C, and 2 µL of the final supernatant was mixed with 2 µL of reservoir solution onto a coverslip in order to crystallize by the hanging drop method, the reservoir solution being as previously reported (3 M $(NH_4)_2SO_4$, Aldrich, #A2939; 50 mM Tris, pH = 8.5; 2 mM PCMB, Sigma, #55540)[8]. Crystals were harvested using a Nylon loop after growing for 5–7 days and frozen in liquid nitrogen after soaking for 5–10 seconds in 25–30% glycerol in 2 M $(NH_4)_2SO_4$ serving as a cryoprotectant.

5.2. Determination of half-maximal inhibitory concentration (IC_{50})

Approximately 1.0 mg of hCAII was dissolved in 100 µL of 50 mM Tris (pH 7.4) and diluted to a final concentration of 500 nM (concentration was determined in duplicates by absorbance spectroscopy) and mixed with the appropriate amount of blocker. The enzyme-blocker solution was then pipetted into a 96-well plate (white, clear flat bottom) containing *p*NPA for a final concentration of 5 mM. This was added simultaneously with a multi-well-pipette. After 20–30 min of incubation in the dark, the absorbance was measured using a plate reader at λ = 400 nm. Obtained data was background substracted and divided by data points of non-inhibited hCAII in order to obtain relative activity. The data points were fitted sigmoidal (eq. 1) or using the Hill equation (eq. 2) using IgorPro (version 6.22A) and the resulting equation was solved to obtain the IC_{50} by using y = 0.5. All experiments were performed at least in triplicates.

$$y = base + [max / (1 + \exp[(xhalf - x)/rate)]] \qquad \text{(eq. 1)}$$

$$y = base + (max - base)/(1 + (xhalf / x)^{rate}) \qquad \text{(eq. 2)}$$

5.3. Determination of inhibitory constants (K_i)

Inhibitory constants were determined using the Cheng–Prussow equation (eq. 3) [9] with the obtained half-maximal inibitory constants (IC_{50}) and with substrate concentration [S] = 5.0 mM.

$$K_i = IC_{50} / (1 + [S]/K_m) \qquad \text{(eq. 3)}$$

The Michaelis–Menten constant K_m was obtained for this assay system independently and was determined to be 1092.5 µM (see Supplementary Figure 1).

6. *Supplementary Figures*

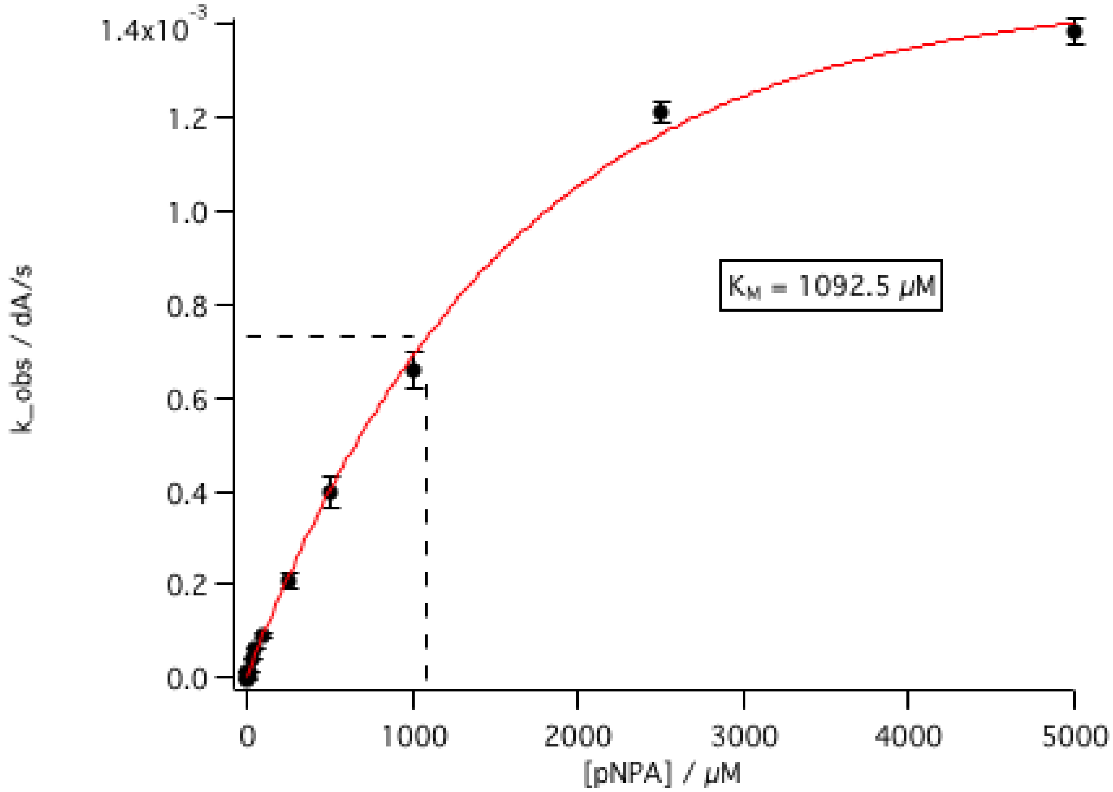

Supplementary Figure 1: Determination of the Michaelis-Menten constant K_M.

7. References

1. Broichhagen, J.; Schönberger, M.; Cork, S. C.; Frank, J. A.; Marchetti, P.; Bugliani, M.; Shapiro, A. M.; Trapp, S.; Rutter, G. A.; Hodson, D. J.; Trauner, D., *Nat Commun* **2014,** *5*, 5116.
2. Carta, F.; Maresca, A.; Scozzafava, A.; Vullo, D.; Supuran, C. T., *Bioorg Med Chem* **2009,** *17* (20), 7093-9.
3. Kabsch, W., *Acta Crystallogr D Biol Crystallogr* **2010,** *66* (Pt 2), 133-44.
4. Vagin, A. A.; Steiner, R. A.; Lebedev, A. A.; Potterton, L.; McNicholas, S.; Long, F.; Murshudov, G. N., *Acta Crystallogr D Biol Crystallogr* **2004,** *60* (Pt 12 Pt 1), 2184-95.
5. Sjoblom, B.; Polentarutti, M.; Djinovic-Carugo, K., *Proc Natl Acad Sci U S A* **2009,** *106* (26), 10609-13.
6. Turk, D., *Acta Crystallogr D Biol Crystallogr* **2013,** *69* (Pt 8), 1342-57.
7. Monnard, F. W.; Heinisch, T.; Nogueira, E. S.; Schirmer, T.; Ward, T. R., *Chem Commun (Camb)* **2011,** *47* (29), 8238-40.
8. Lesburg, C. A.; Huang, C.; Christianson, D. W.; Fierke, C. A., *Biochemistry* **1997,** *36* (50), 15780-91.
9. Cheng, Y.; Prusoff, W. H., *Biochem Pharmacol* **1973,** *22* (23), 3099-108.
